# MORC1 coordinates TRIM28 interaction and nuclear condensate formation for transposon silencing

**DOI:** 10.1016/j.isci.2026.117394

**Published:** 2026-08-31

**Authors:** Yosuke Isota, Peilin Li, Kiichiro Sakata, Hiyu Chiwata, Hidetaka Kosako, Soichiro Yamanaka, Shinsuke Ito, Hiroya Yamazaki, Mikiko C. Siomi

**Affiliations:** 1 Department of Biological Sciences, Graduate School of Science, The University of Tokyo, Tokyo 113-0032, Japan; 2 Division of Cell Signaling, Fujii Memorial Institute of Medical Sciences, Tokushima University, Tokushima 770-8503, Japan; 3 Center for Integrative Medical Sciences, RIKEN, Kanagawa 230-0045, Japan

**Keywords:** MORC1, TRIM28, gonocytes, intrinsically disordered region, nuclear condensate, transposon repression

## Abstract

MORC1-mediated transposon repression involves DNA methylation and H3K9me3 deposition, yet the underlying mechanism remains unclear. Here, we show that MORC1 assembles discrete nuclear bodies in gonocytes and, using a doxycycline-inducible NIH3T3 cell system, define two properties: MORC1 drives nuclear condensate formation through an intrinsically disordered region (IDR) containing a bipartite nuclear localization signal, and MORC1 binds TRIM28, a corepressor that recruits the H3K9 methyltransferase SETDB1, and concentrates it within these bodies through protein-protein interactions. An IDR-deficient MORC1 mutant fails to form nuclear condensates but retains TRIM28 binding. Chromatin immunoprecipitation sequencing (ChIP-seq) reveals that this mutant occupies heterochromatin-embedded transposons more strongly than wild-type MORC1, particularly evolutionarily young subfamilies overlapping endogenous MORC1 targets. Notably, exogenous MORC1 forms substantially larger condensates than endogenous MORC1 in gonocytes, and fluorescence recovery after photobleaching (FRAP) analysis shows that MORC1 condensates limit diffusion. We propose a model in which MORC1 function is shaped by coordinated TRIM28 interaction and quantitatively restrained, IDR-dependent condensate formation.

## Highlights

• MORC1 forms nuclear condensates through an intrinsically disordered region
• MORC1 condensates restrain diffusion and regulate chromatin function
• MORC1 binds TRIM28 and sequesters it within nuclear condensates
• MORC1 coordinates its own targeting and that of TRIM28 to young LINE-1 elements

## Graphical abstract

**Figure:**
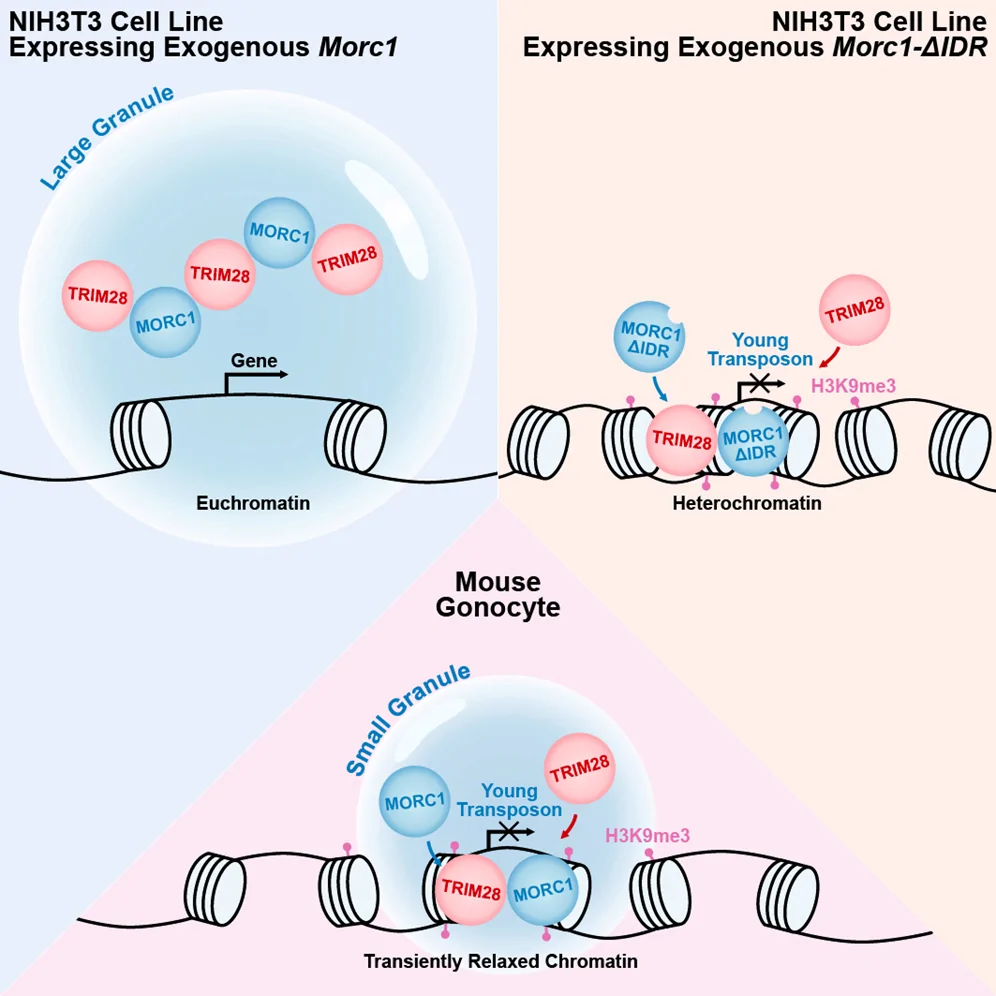

## Teaser

Natural sciences; Biological sciences; Genomics; Molecular biology; Epigenetics; Cell biology; Bioinformatics; Genomic analysis

## Introduction

Microrchidia family CW-type zinc finger (MORC) proteins are evolutionarily conserved across diverse organisms, from prokaryotes to eukaryotes.^1^ In humans, four paralogs, MORC1−MORC4, have been identified.^2^ The mouse genome encodes five paralogs, MORC1, MORC2a, MORC2b, MORC3, and MORC4, each associated with distinct biological functions.^3^^,^^4^^,^^5^^,^^6^^,^^7^ Among them, MORC2a is a GHKL ATPase that functions as an effector of the HUSH complex, contributing to heterochromatin compaction and gene silencing.^8^ MORC2b, a rodent-specific paralog of MORC2a, regulates the meiotic program,^5^ whereas MORC3 contributes to transposon silencing in mouse embryonic stem cells.^6^ By contrast, the function of MORC4 remains poorly understood. MORC1 is unique in that it plays a fertility-specific role: its deficiency causes male infertility, whereas no detectable effects have been observed on female reproductive function.^3^^,^^9^^,^^10^^,^^11^ Notably, MORC-mediated transposon silencing is also conserved in plants,^12^^,^^13^^,^^14^ where MORC proteins function in association with the RNA-directed DNA methylation pathway. However, unlike in mice, disruption of plant MORC proteins is not generally associated with overt infertility.

MORC1 is highly expressed in mouse gonocytes, fetal male germ cells present from embryonic day 13.5 (E13.5) to postnatal day 3 (P3).^11^ In these cells, genomic DNA methylation is progressively established by *de novo* DNA methylation, reaching a robust level around P0.^15^ Failure to reestablish DNA methylation leads to persistent transposon activation, which disrupts meiosis by inducing DNA double-strand breaks.^16^ Transposon silencing in gonocytes also involves histone modifications, such as H3K9 trimethylation (H3K9me3), in addition to DNA methylation.^11^ In mice, these epigenetic mechanisms are mediated primarily by two protein families: Krüppel-associated box domain zinc finger proteins (KRAB-ZFPs) and PIWI proteins.^17^^,^^18^

KRAB-ZFPs bind specific transposon-derived genomic sequences and recruit the scaffold protein tripartite motif-containing 28 (TRIM28; also known as KRAB-associated protein 1 [KAP1]), which facilitates chromatin remodeling via interaction with factors such as the H3K9 methyltransferase SETDB1, the *de novo* DNA methyltransferase DNMT3, and the NuRD complex that mediates histone H3K9 deacetylation.^19^^,^^20^^,^^21^^,^^22^ These modifiers establish repressive chromatin states and DNA methylation at transposon loci. The KRAB-ZFP family has rapidly expanded in mammals, likely in response to evolving transposon threats.^23^^,^^24^

PIWI proteins, loaded with PIWI-interacting RNAs (piRNAs), assemble piRNA-induced silencing complexes (piRISCs) that target transposon transcripts via RNA-RNA base pairing.^25^ In mice, two PIWI proteins, MILI (PIWIL2) and MIWI2 (PIWIL4), are active in gonocytes, functioning in the cytoplasm and the nucleus, respectively.^26^^,^^27^ MIWI cleaves transposon transcripts in the cytoplasm, whereas MIWI2 suppresses transposons through transcriptional silencing involving DNA methylation and histone modification.^28^ When a new transposon invades the germline genome, novel piRNAs are generated against the new element.^29^ Thus, like KRAB-ZFPs, the PIWI-piRNA pathway also evolves in response to newly emerging transposons, reflecting an ongoing arms race.

We recently showed that transposons derepressed in MORC1-deficient gonocytes largely overlap with those derepressed in MIWI2 mutants, suggesting that MORC1 contributes to MIWI2-dependent transposon silencing.^11^ In parallel, H3K9me3 levels decline at transposon loci in MORC1-deficient germ cells, accompanied by chromatin opening, supporting a model in which MORC1 promotes heterochromatin formation by re-establishing H3K9me3 at a subset of transposons.^11^ However, detailed mechanistic analysis has been lacking, in part because MORC1 expression is restricted to fetal male germ cells.

In this study, we first immunostained mouse gonocytes with anti-MORC1 antibodies and found that MORC1 forms discrete nuclear condensates. Because detailed analysis of these structures is technically challenging in gonocytes, we established a doxycycline (Dox)-inducible MORC1 expression system in NIH3T3 cells. Although NIH3T3 cells lack the PIWI pathway, they retain the core gene-regulatory machineries, providing a tractable platform for mechanistic analyses.

## Results

### MORC1 assembles nuclear condensates via IDR-mediated phase separation

**Figure 1:**
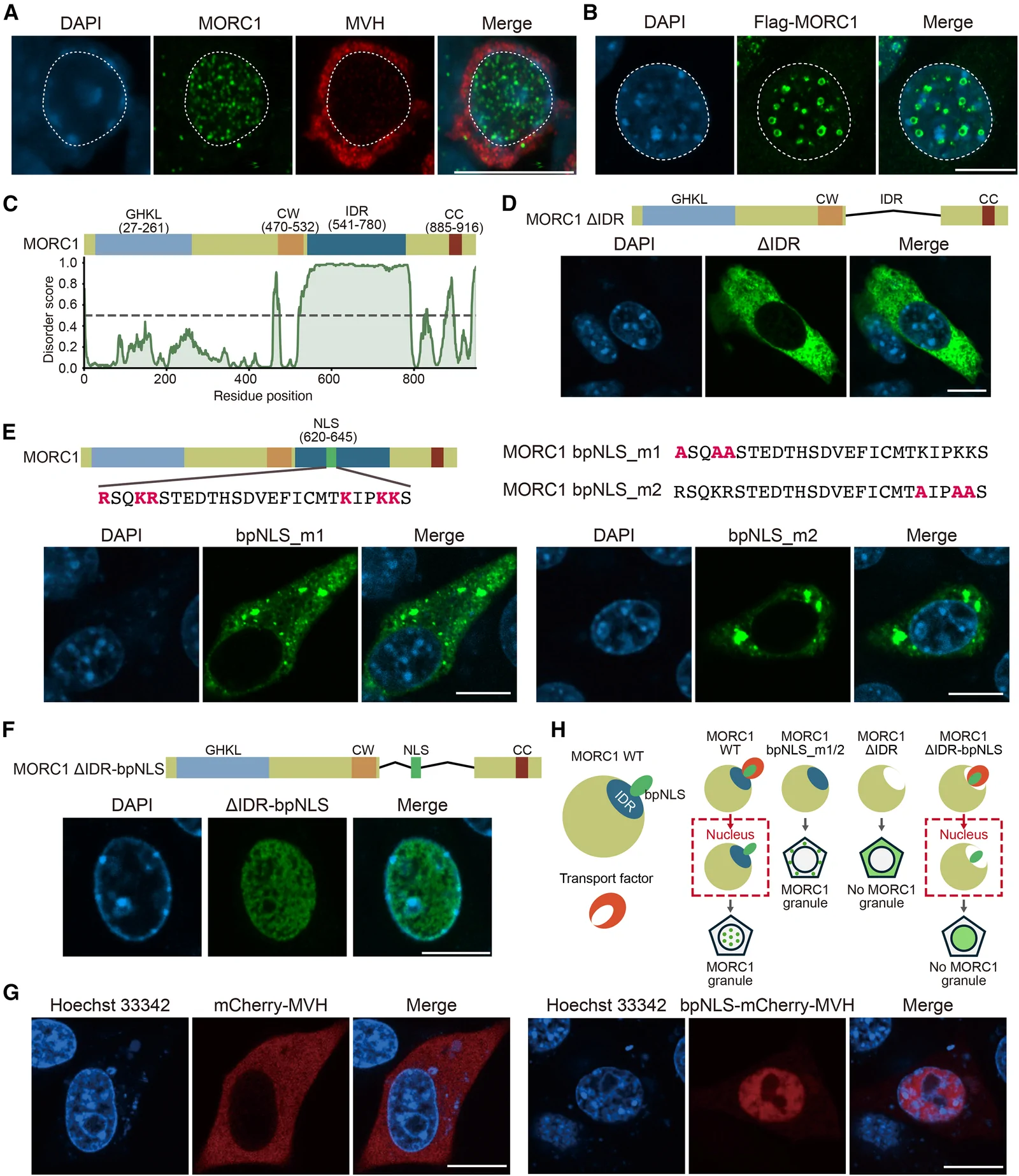
MORC1 assembles nuclear condensates through an IDR containing a bpNLS (A) Endogenous MORC1 (green) forms discrete nuclear foci (“MORC1 bodies”) in MVH-positive (red) gonocytes in the E17.5 fetal testis. Scale bars, 10 μm. (B) Upon Dox induction, FLAG-MORC1 (green) forms nuclear condensates in NIH3T3 cells. Nuclei (white dotted outline) stained with DAPI (blue). Scale bars, 10 μm. (C) Schematic of MORC1 domain architecture and predicted IDR (blue, aa 541–780) immediately downstream of the CW domain (orange, aa 470–532). GHKL domain (gray, aa 27–261); coiled-coil (CC) domain (brown, aa 885–916). (D) MORC1 ΔIDR (green) is dispersed throughout the cytoplasm in NIH3T3 cells. Nuclei stained with DAPI (blue). Scale bars, 10 μm. (E) bpNLS mutants (green) exhibit cytoplasmic localization. bpNLS_m1: Arg620, Lys623, and Arg624 were substituted with alanine; bpNLS_m2: Lys640, Lys643, and Lys644 were substituted with alanine. Nuclei stained with DAPI (blue). Scale bars, 10 μm. (F) The MORC1 ΔIDR-bpNLS mutant (green) localizes to the nucleus but fails to form condensates. Nuclei stained with DAPI (blue). Scale bars, 10 μm. (G) Fusion of the MORC1 bpNLS to mCherry-MVH (red) redirects the fusion protein to the nucleus. Nuclei stained with Hoechst 33342 (blue). Scale bars, 10 μm. (H) Intracellular localization and dynamics of MORC1 (WT and mutants).

To assess the endogenous behavior of MORC1, we immunostained fetal male germ cells with anti-MORC1 antibodies. MORC1 was detected in mouse Vasa homolog (MVH)-positive cells (Figures 1A and S1A), where it formed discrete nuclear foci (“MORC1 bodies”) with a median estimated area of 0.043 μm^2^ (Figure S1B). MVH is a germ-cell-specific RNA helicase that is widely used as a marker for identifying germ cells.^30^ A previous study reported that MORC1 is distributed more diffusely throughout the nucleus in mouse gonocytes.^3^ This difference may reflect differences in the antibodies used between the studies. Given the emerging importance of phase separation in chromatin dynamics and gene regulation,^31^^,^^32^^,^^33^^,^^34^ our finding that MORC1, a germline-specific transposon silencing factor, forms condensates further supports the idea that phase separation contributes to genome regulation in germ cells.

Because further multifaceted analysis of these condensates is technically challenging in gonocytes, we generated a Dox-inducible FLAG-MORC1 line in NIH3T3 cells. Short-read RNA-seq^35^ showed that NIH3T3 endogenously express *Morc2a* (NCBI gene ID: 74522), *Morc3* (338467), and *Morc4* (75746) but not *Morc1* (17450) or *Morc2b* (240069) (Figures S1C and S1D). In contrast, RNA-seq data of male germ cells^11^^,^^36^^,^^37^ indicated high *Morc1* expression in gonocytes, followed by *Morc3* and *Morc2a*, with little or no *Morc2b* and *Morc4* (Figure S1E).

Upon Dox induction, anti-FLAG immunostaining revealed prominent MORC1 nuclear condensates that were substantially larger than MORC1 bodies in gonocytes (median area, 0.144 μm^2^) (Figures 1B and S1B; 100 ng/mL for 48 h). Signals were sometimes reduced in the condensate center, producing a ring-like morphology, consistent with antibody exclusion from dense phases or with proteins known to undergo phase separation.^38^ Condensates frequently neighbored DAPI-bright structures (Figure 1B), likely constitutive heterochromatin or the nucleolus,^39^ although the functional relevance of this proximity remains unclear.

MORC1 bodies in gonocytes were much smaller and lacked a ring-like morphology (Figures 1A, 1B, and S1B). To investigate whether the observed size difference could be attributed to MORC1 expression levels, we conducted a titration of Dox-induced MORC1 expression in NIH3T3 cells. Reducing the induction period alone (100 ng/mL for 24 h; median area 0.104 μm^2^) did not significantly alter condensate size compared to the standard condition (100 ng/mL for 48 h). Decreasing the Dox concentration to 10 ng/mL for 24 h resulted in a reduction of the median condensate area to 0.087 μm^2^, although this value remained approximately 2.0 times larger than that of gonocyte condensates. Further reduction to 1 ng/mL Dox for 24 h eliminated discernible condensate formation (Figure S1F). These findings suggest that the smaller size of condensates in gonocytes cannot be attributed solely to reduced MORC1 expression. Rather, germ-cell-specific factors, such as nuclear architecture, chromatin environment, or cell-type-specific interaction partners, may also contribute to the regulation of condensate properties *in vivo*.

Because many condensate-forming proteins rely on intrinsically disordered regions (IDRs), we queried MORC1 with DISOPRED3.^40^ This identified a predicted IDR immediately C-terminal to the CW domain (Figures 1C and S1G). We deleted this segment and expressed the mutant (MORC1 IDR deletion [ΔIDR]) in NIH3T3 cells. In contrast to wild-type (WT) MORC1, the ΔIDR mutant failed to form nuclear foci and instead displayed a diffuse cytoplasmic distribution (Figure 1D), indicating that the IDR is required for nuclear condensate assembly and likely contributes nuclear localization.

### The MORC1 IDR harbors a bipartite NLS required for nuclear granule assembly and suppresses cytoplasmic granules

We queried the MORC1 IDR for nuclear localization signal (NLS) using cNLS Mapper^41^^,^^42^^,^^43^ and identified a canonical bipartite NLS (bpNLS) spanning Arg620–Ser645 (Figures 1E and S1G). The motif contains two basic clusters: Arg620–Arg624 near the N-terminal side and Lys640–Lys644 toward the C-terminal side.

To test function, we alanine-substituted three basic residues in either cluster, Arg620/Lys623/Arg624 (bpNLS_m1) or Lys640/Lys643/Lys644 (bpNLS_m2). Both mutants lost nuclear localization and instead formed cytoplasmic aggregates (Figure 1E), indicating that the predicted region serves as a *bona fide* bpNLS and that nuclear import, while necessary for nuclear granules, is not required for condensate assembly per se.

We next restored the bpNLS to the IDR-deleted mutant (ΔIDR-bpNLS). The fusion localized to nuclei but failed to assemble MORC1 granules (Figure 1F), showing that the IDR is indispensable for condensate formation, whereas the bpNLS is sufficient only for import. Fusing the MORC1 bpNLS to a predominantly cytoplasmic MVH (mCherry-MVH) drove its nuclear accumulation, whereas mCherry-MVH alone remained cytoplasmic (Figure 1G), demonstrating that the MORC1 bpNLS is sufficient to confer nuclear localization activity.

These results support a model (Figure 1H) in which a transport factor engages the MORC1 bpNLS to promote nuclear import, thereby preventing MORC1 from accumulating in the cytoplasm, where it would otherwise form condensates. Notably, an analogous mechanism has been reported for the spindle assembly factor TPX2.^44^

To examine the biophysical characteristics of MORC1 condensates, we conducted fluorescence recovery after photobleaching (FRAP) analysis. For WT MORC1, individual condensates were photobleached, whereas for ΔIDR-bpNLS, which does not form discernible condensates, nuclear regions of equivalent area were bleached. WT MORC1 demonstrated significantly slower fluorescence recovery than ΔIDR-bpNLS, with median recovery time constants of 37.8 s and 5.27 s, respectively (Figures S1H and S1I). This pronounced difference suggests that IDR-dependent condensate formation markedly restricts MORC1 molecular mobility, thereby diminishing the freely diffusible pool of MORC1 available for genomic surveillance.

### MORC1 binds and concentrates TRIM28 into MORC1 granules

**Figure 2:**
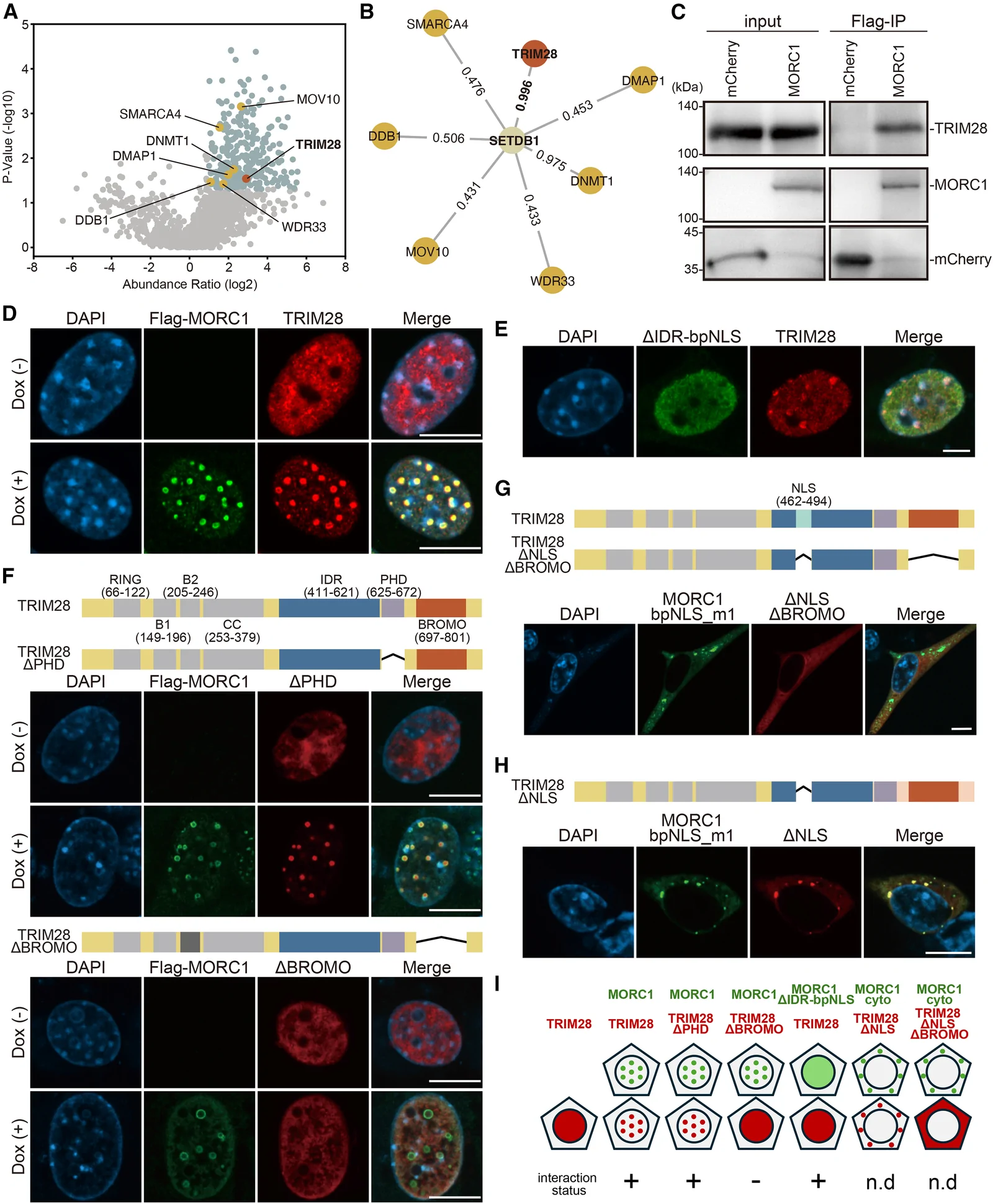
MORC1 binds and concentrates TRIM28 into MORC1 condensates (A) Volcano plot showing proteins co-immunoprecipitating with MORC1 in NIH3T3 cells. Proteins appearing in (B) are specified by protein names. TRIM28 is shown in red. (B) STRING-based interaction network with SETDB1 as bait. The combined STRING scores are indicated on the edges. (C) Co-immunoprecipitation of exogenously expressed MORC1 and TRIM28 in NIH3T3 cells. (D) TRIM28 (red) is diffusely distributed in the nucleoplasm of Dox (−) NIH3T3 cells but colocalizes with MORC1 (green) upon its expression (Dox (+)). Nuclei stained with DAPI (blue). Scale bars, 10 μm. (E) TRIM28 (red) remains nucleoplasmic when MORC1 ΔIDR-bpNLS (green) is expressed instead of WT in (D). Nuclei stained with DAPI (blue). Scale bars, 10 μm. (F) The TRIM28 ΔPHD mutant (red) is efficiently reposited into MORC1 condensates (green), whereas the ΔBROMO mutant (red) fails to localize to them. Nuclei stained with DAPI (blue). Scale bars, 10 μm. (G) TRIM28 ΔNLSΔBROMO (red) and MORC1-bpNLS_m1 (green) fail to colocalize. Nuclei stained with DAPI (blue). Scale bars, 10 μm. (H) TRIM28 ΔNLS (red) and MORC1-bpNLS_m1 (green) colocalize in distinct cytoplasmic foci. Nuclei stained with DAPI (blue). Scale bars, 10 μm. (I) Summary of immunofluorescence assays.

We profiled MORC1-interacting proteins in NIH3T3 cells. Following Dox-induced expression of FLAG-MORC1, anti-FLAG immunoprecipitates were analyzed by mass spectrometry (MS) (Table S1). Volcano plots from three biological replicates (Figure S2A) revealed numerous proteins enriched in MORC1 immunoprecipitates (Figure 2A). To prioritize candidates, we overlaid STRING interaction scores using SETDB1, the H3K9me3 methyltransferase for transposon silencing, in gonocytes as a network anchor.^45^^,^^46^ TRIM28 emerged among the top hits with high STRING confidence (combined STRING score: 0.996) and strong MS enrichment (log_2_ abundance ratio >2.9; -log_10_ *p* > 1.5) (Figures 2A and 2B). TRIM28 and additional network-supported factors are highlighted in Figures 2A and 2B. MORC1 robustly co-precipitated with TRIM28, whereas the negative control (mCherry) did not (Figure 2C), supporting the placement of MORC1 within the TRIM28/SETDB1 axis.

Immunofluorescence showed that, in naive NIH3T3 cells lacking MORC1 induction, endogenous TRIM28 was mainly nucleoplasmic (Dox (-); Figure 2D). Upon MORC1 induction, TRIM28 sharply colocalized within MORC1 nuclear granules (Dox (+)), indicating that MORC1 recruits TRIM28 into MORC1 condensates through protein-protein interaction.

### TRIM28 bromodomain mediates TRIM28-MORC1 association

Replacing WT MORC1 with the IDR-deleted mutant (ΔIDR-bpNLS) abolished TRIM28 concentration: TRIM28 remained largely nucleoplasmic as in MORC1-deficient cells (Figure 2E). Notably, ΔIDR-bpNLS still co-immunoprecipitated endogenous TRIM28 (Figure S2B), indicating that MORC1-TRIM28 binding is separable from MORC1 granule formation.

To delineate the TRIM28 region required for recruitment, we generated ΔPHD (PHD-domain deletion) and ΔBROMO (bromodomain deletion) mutants (Figure S2C). The PHD domain promotes SUMOylation of the adjacent bromodomain, which then recruits repressors such as SETDB1 to silence targets, including transposons.^47^ Upon co-expression with WT MORC1, ΔPHD robustly concentrated into MORC1 granules, whereas ΔBROMO failed to localize to granules and remained diffusely nuclear (Figure 2F). Consistently, co-immunoprecipitation (coIP) detected MORC1 interaction with ΔPHD but not with ΔBROMO (Figure S2D), indicating that the bromodomain is essential for both MORC1 interaction and granule recruitment. TRIM28 also harbors a bpNLS within its IDR^48^ (Glu462–Asp494; Figures 2G and S2C). A ΔBROMO mutant lacking this NLS failed to co-aggregate with cytoplasmic MORC1-bpNLS_m1 (Figure 2G). However, restoring the bromodomain enabled a ΔNLS mutant to colocalize with MORC1-bpNLS_m1 (Figure 2H), further supporting the requirement for the bromodomain in MORC1-dependent granule recruitment. Figure 2I summarizes subcellular localization patterns from these immunofluorescence assays and the MORC1-TRIM28 interaction status.

### CW domain dictates family-specific TRIM28 concentration

**Figure 3:**
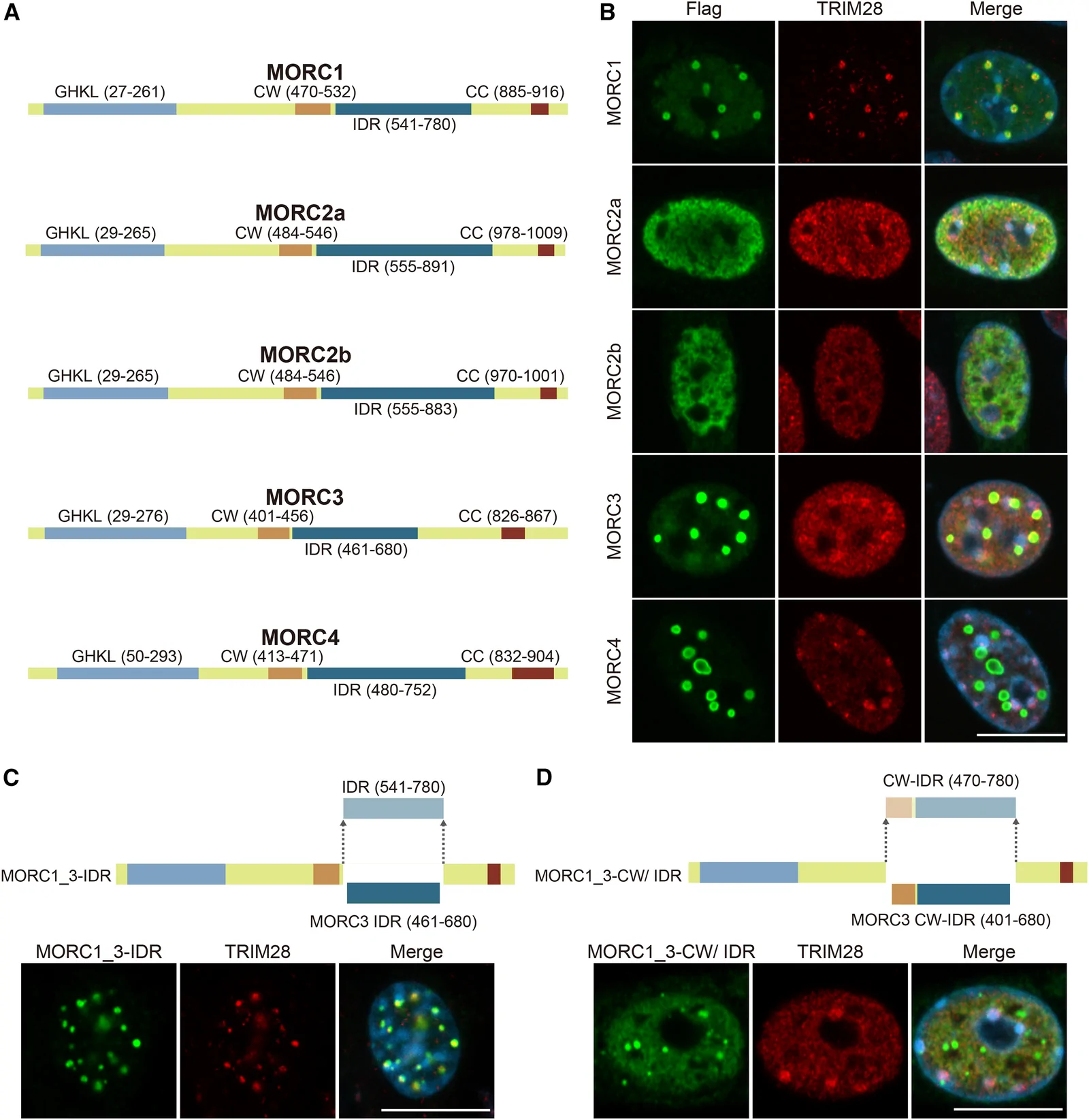
MORC1 exerts the strongest effect on the subcellular localization of TRIM28 among MORC family members (A) Domain organization of MORC family members. The IDR of each member were determined based on their amino acid sequence homology with MORC1 IDR. (B) Subcellular localization of MORC1 family members (green) in NIH3T3 cells. TRIM28 is shown in red. Nuclei stained with DAPI (blue). Scale bars, 10 μm. (C) The MORC1_3-IDR chimera (green) concentrates TRIM28 (red) into nuclear condensates. Nuclei stained with DAPI (blue). Scale bars, 10 μm. (D) The MORC1_3-CW/IDR chimera (green) fails to concentrate TRIM28 (red) into the condensates. Nuclei stained with DAPI (blue). Scale bars, 10 μm.

Although MORC family members share a similar overall domain architecture (Figure 3A), their behaviors in NIH3T3 cells were clearly distinct. MORC1, MORC3, and MORC4 formed nuclear foci, with MORC3 and MORC4 tending to form larger structures, whereas MORC2a and MORC2b were distributed predominantly throughout the nucleoplasm (Figure 3B). Nuclear body formation has previously been reported for human MORC3.^49^^,^^50^ In addition, nematode MORC-1, which is implicated in nuclear RNAi and chromatin compaction, has been shown to form nuclear bodies through multimeric assemblies mediated by dimerization and binding to DNA loops.^51^^,^^52^

We next compared how each MORC alters TRIM28 localization (Dox (-), Figure 2D). MORC2a, MORC2b, and MORC4 had minimal effect; MORC3 partially shifted TRIM28 into foci; MORC1 produced the strongest accumulation (Figures 3B and S3A). Of the family, MORC2a, MORC3, and MORC4 are endogenously expressed in naive NIH3T3 (Figure S1D), consistent with their limited impact on TRIM28 (MORC3 modest).

Because the MORC1 IDR is required for granule formation, we asked whether domain differences explain functional divergence. Replacing the MORC1 IDR with that of MORC3 (MORC1_3-IDR) preserved TRIM28 reposition, comparable to WT MORC1 (Figure 3C). In contrast, swapping both the CW domain and IDR (MORC1_3-CW/IDR) markedly reduced concentration, phenocopying WT MORC3 despite robust nuclear foci (Figure 3D). The CW zinc finger is a chromatin reader^53^^,^^54^; sequence identity between MORC1 and MORC3 CWs is low (31.7%; Figure S3B), and the MORC3 CW preferentially binds H3K4me3, a mark of active genes.^6^^,^^55^^,^^56^

These data indicate that while IDR licenses condensate formation, the CW domain is the principal determinant of family specificity and governs interactions with other modules. Swapping in the CW domain of MORC3 retargets MORC1 to H3K4me3-marked chromatin while concomitantly weakening its interaction with TRIM28. This accounts for the attenuated TRIM28 recruitment observed with MORC1_3-CW/IDR. In this context, active marker-guided chromatin recognition either competes with or dilutes TRIM28 binding.

### IDR-dependent control of MORC1 genomic occupancy

**Figure 4:**
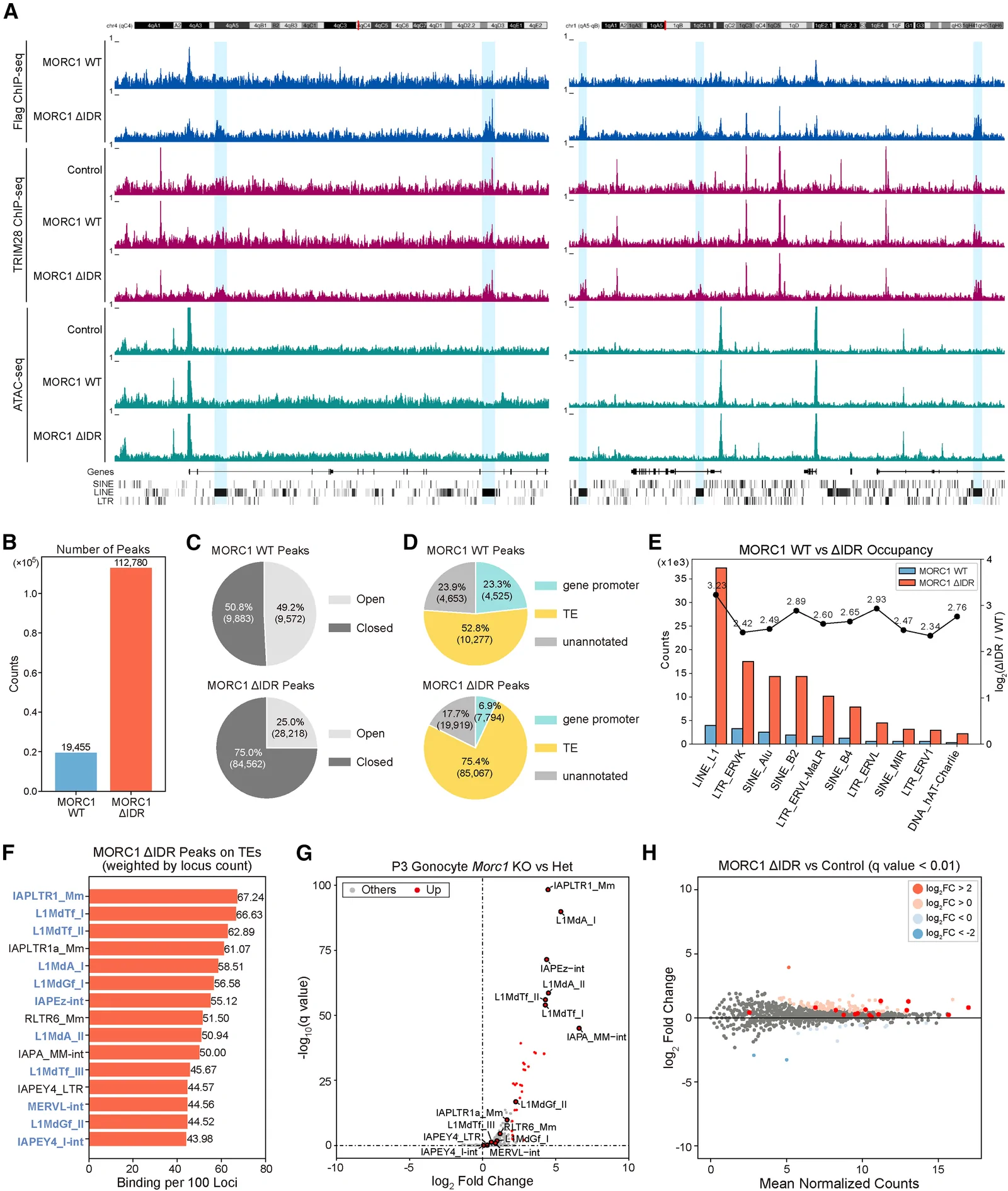
MORC1 preferentially associates with evolutionarily young transposons (A) Genome browser snapshots showing MORC1 and TRIM28 ChIP-seq and ATAC-seq profiles. Signal tracks were generated from merged BAM files of two biological replicates for each condition. MORC1 ΔIDR, ΔIDR-bpNLS mutant; control, mCherry. Blue highlights mark MORC1 peaks identified specifically in the ΔIDR mutant relative to WT. (B) Number of MORC1 ChIP-seq peaks detected in WT (blue) and ΔIDR (red) samples. (C) Distribution of MORC1 ChIP-seq peaks in open or closed chromatin regions defined by ATAC-seq peaks. Upper, WT; lower, ΔIDR. (D) Genomic annotation of MORC1 ChIP-seq peaks as gene promoter, transposon (TE), or unannotated. Upper, WT; lower, ΔIDR. (E) Bar plot showing the number of MORC1 WT and ΔIDR peaks overlapping each TE family, sorted by total peak occupancy across both conditions (left *y* axis, counts). Line plot showing the log_2_ ratio of peak occupancy in ΔIDR relative to WT for each TE family (right *y* axis, log_2_(ΔIDR/WT)). (F) Top 15 TE subfamilies most frequently occupied by MORC1 ΔIDR peaks, with binding frequency normalized per 100 loci. Evolutionarily young subfamilies (Figure S4M) are highlighted in blue. (G) Volcano plot of TE expression changes in P3 gonocytes from *Morc1* knockout (KO) and heterozygous (Het) mice. Significantly upregulated TE subfamilies are highlighted in red, and subfamilies listed in (F) are annotated. (H) MA plot showing expression changes in TE subfamilies between NIH3T3 cells expressing MORC1 ΔIDR and control (mCherry). Subfamilies with *q*.value <0.01 and log_2_FC >2, >0, <0, or < -2 are annotated as indicated in the legend. Subfamilies listed in (F) are highlighted in bright red.

Nuclear colocalization of MORC1 and TRIM28, together with their protein-protein interaction, suggests that they may co-occupy genomic loci. To test this, we performed chromatin immunoprecipitation followed by sequencing (ChIP-seq) in NIH3T3cells expressing FLAG-MORC1 WT, using anti-FLAG and anti-TRIM28 antibodies. Because the ΔIDR-bpNLS mutant failed to form nuclear condensates (Figure 1F) but retained TRIM28 binding at levels comparable to WT (Figure S2B), we also analyzed this mutant (Figure 4A). To assess how the chromatin landscape changes in the presence or absence of MORC1, we additionally performed assay for transposase-accessible chromatin using sequencing (ATAC-seq) in NIH3T3 cells expressing FLAG-mCherry (control), FLAG-MORC1 WT, or the ΔIDR-bpNLS mutant (Figures 4A and S4A–S4C).

We first compared the ChIP-seq datasets for WT and mutant MORC1. Peak calling identified 19,455 peaks for WT and 112,780 peaks for the ΔIDR-bpNLS mutant (Figure 4B), even though WT and mutant MORC1 were expressed at comparable levels (Figure S4D). These results suggest that IDR-dependent condensate formation restricts MORC1 genomic occupancy.

Comparison with ATAC-seq datasets revealed that 50.8% of WT peaks (*n* = 9,883) and 75.0% of mutant peaks (*n* = 84,562) fall within closed chromatin (Figure 4C). This suggests reduced access of WT MORC1 to heterochromatic regions. By contrast, global chromatin accessibility measured by ATAC-seq was largely unchanged between WT- or mutant-expressing cells (Figure S4E). Thus, in this context, exogenous MORC1 binding to NIH3T3 chromatin appears insufficient to remodel the pre-established chromatin landscape.

Almost all transposons in NIH3T3 cells reside in closed chromatin (98.3%; Figure S4F). Consistent with this, 75.4% of ΔIDR-bpNLS peaks (*n* = 85,067) mapped within transposon sequences, whereas 52.8% of WT peaks (*n* = 10,277) did so (Figure 4D). We next quantified the number of transposon loci harboring WT or mutant MORC1 peaks, identifying 17,508 and 119,105 transposons, respectively (Figure S4G). The overlap was asymmetric: 68.4% of WT-bound transposons were also bound by the mutant, whereas only 10.0% of mutant-bound transposons overlapped with WT. This is consistent with restricted access of WT MORC1 to heterochromatin-embedded transposons.

Although less prominent, MORC1, particularly WT, also showed appreciable binding to gene promoters (23.3%; Figure 4D). To identify which genes are preferentially targeted by MORC1 WT, we examined its promoter-bound genes and found enrichment for genes involved in RNA processing and/or translation, many of which are housekeeping genes (Figure S4H). The mutant exhibited reduced promoter binding (6.9%; Figure 4D) but was associated with similar functional categories (Figure S4I). This bias, regardless of IDR presence, suggests that MORC1 does not bind the genome in a purely stochastic manner.

To examine whether MORC1 expression and chromatin binding are associated with changes in gene expression, we performed RNA-seq in NIH3T3 cells expressing mCherry, WT MORC1, or the ΔIDR-bpNLS mutant (Figure S4J). We then classified MORC1-bound genes into WT-unique, mutant-unique, and shared (common) categories (Figure S4K). This analysis showed that, in NIH3T3 cells, MORC1 promoter binding, whether by WT MORC1 or the IDR-lacking mutant, is largely uncoupled from detectable changes in steady-state gene expression (Figure S4L). Consistently, promoter-proximal chromatin features were broadly unchanged, with no evident differences in TSS-centered chromatin accessibility, as assessed by ATAC-seq across conditions (Figure S4L). These observations indicate that promoter association alone has limited transcriptional consequences in NIH3T3 cells.

### MORC1 preferentially binds young LINE_L1 and LTR_ERVK elements

Because the number of MORC1-bound transposons was substantially higher in cells expressing ΔIDR-bpNLS mutant (*n* = 119,105) than in those expressing WT MORC1 (*n* = 17,508), we next compared WT and mutant occupancy across transposon classes (Figure 4E). This analysis revealed that the ΔIDR-bpNLS mutant was more broadly enriched than WT MORC1 across multiple transposon classes, with the strongest enrichment observed at LINE_L1 elements (log_2_(mutant/WT) = 3.23; Figure 4E). Subfamily-level analysis further showed that ΔIDR-bpNLS peaks were preferentially enriched at evolutionarily young LINE_L1 and LTR_ERVK subfamilies (Figures 4F and S4M), whereas such enrichment was not observed for random peaks or WT MORC1 peaks (Figures S4M−S4P).

We previously identified transposons that are derepressed in MORC1-deficient gonocytes.^11^ We, therefore, compared these elements with those bound by exogenously expressed MORC1 in NIH3T3 cells. Notably, the ΔIDR-bpNLS mutant, but not WT MORC1, showed a striking overlap with these derepressed, evolutionarily young elements (Figures 4F, 4G, S4O, and S4Q). This observation suggests that MORC1 tends to target a similar subset of transposons, whether expressed endogenously in gonocytes or ectopically in NIH3T3 cells.

Despite clear occupancy at transposon loci, particularly by the ΔIDR-bpNLS mutant, MORC1 binding had little effect on transposon expression under either the WT or ΔIDR-bpNLS condition (Figures 4H and S4R). This limited transcriptional effect is likely because most transposons in NIH3T3 cells already reside within strongly repressed heterochromatin.

### MORC1 modulates TRIM28 recruitment to target transposons

**Figure 5:**
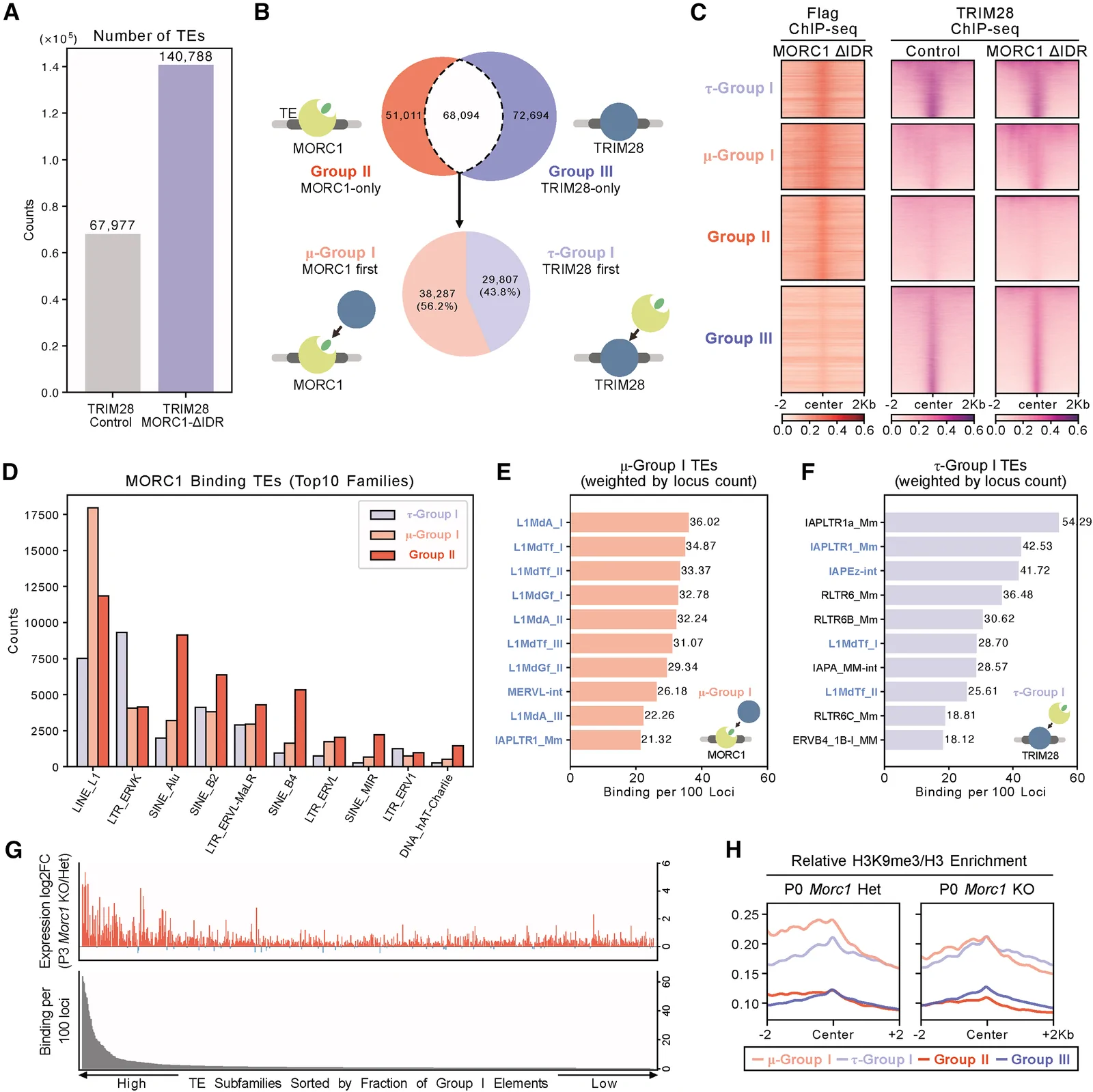
TRIM28 association with MORC ΔIDR at young transposon loci (A) Number of transposons harboring TRIM28 ChIP-seq peaks in control (mCherry; gray) and MORC1 ΔIDR-expressing (purple) samples. (B) Classification scheme for transposons harboring MORC1 ΔIDR peaks and TRIM28 peaks in MORC1-ΔIDR cells. Group I (white), transposons containing both MORC1 ΔIDR and TRIM28 peaks; group II (red), transposons containing MORC1 ΔIDR peaks only; group III (purple), transposons containing TRIM28 peaks only. Group I transposons were further subdivided into μ-group I (MORC1-first; pale red), in which TRIM28 peaks were newly detected in MORC1-ΔIDR cells relative to control cells, and τ-group I (TRIM28-first; pale purple), in which TRIM28 peaks were already present in control cells. (C) Heatmaps showing MORC1 ΔIDR and TRIM28 enrichment across transposons in τ-group I, μ-group I, group II, and group III. Transposons in all heatmaps are sorted by MORC1 ΔIDR enrichment (left). MORC1 ΔIDR expression enhances TRIM28 accumulation at transposon loci of μ-group I. (D) Transposon families most frequently represented among MORC1 binding elements (τ-group I, μ-group I, and group II). (E and F) Top 10 transposon subfamilies within μ-group I (E) and τ-group I (F), with binding frequency normalized per 100 loci. Evolutionarily young subfamilies (Figure S4M) are highlighted in blue. (G) Correlation within transposon subfamilies between the fraction of group I (μ-group I and τ-group I) transposons (bottom) and expression changes in P3 *Morc1*-deficient gonocytes (top). (H) Line plots showing relative H3K9me3 enrichment across τ-group I, μ-group I, group II, and group III transposons in P0 *Morc1* Het and KO gonocytes. Relative H3K9me3 enrichment was calculated as the ratio of H3K9me3 to H3 ChIP-seq signal.

We next analyzed the TRIM28 ChIP-seq datasets and found 67,977 transposons contained TRIM28 peaks in control (mCherry-expressing) cells, whereas 140,788 transposons harbored TRIM28 peaks in MORC1 (ΔIDR-bpNLS)-expressing cells (Figure 5A), representing a 2.1-fold increase in TRIM28-targeted transposons upon expression of MORC1. Thus, ΔIDR-bpNLS expression increased TRIM28 occupancy at transposon loci.

We next asked how many of these TRIM28-bound transposons were also associated with MORC1 (ΔIDR-bpNLS) (Figure S5A). This analysis identified 68,094 transposons co-bound by TRIM28 and MORC1 (group I, white area enclosed by the dotted line in Figure 5B). Among the remainder, 51,011 transposons were bound only by MORC1 (MORC1-only; group II, red), whereas 72,694 were bound only by TRIM28 (TRIM28-only; group III, purple). We further subdivided group I transposons into two categories: loci at which TRIM28 was already bound before MORC1 expression (TRIM28 first; τ-group I) (*n* = 29,807; 43.8%) and loci at which TRIM28 binding appeared concomitantly with, or subsequently to, MORC1 binding (MORC1 first; μ-group I) (*n* = 38,287; 56.2%) (Figure 5B).

We also examined the distribution of MORC1 and TRIM28 across transposons in each group, i.e., τ-group I, μ-group I, group II, and group III (Figure 5C). Heatmaps show that MORC1 exhibited relatively stable enrichment on τ-group I, μ-group I, and group II transposons, whereas, as expected, little enrichment was observed on group III transposons. TRIM28 was substantially recruited to transposons in μ-group I (MORC1 first) (magenta panels in the second row from the top). In contrast, only minor changes were observed in τ-group I, group II, and group III. These data defined distinct classes of MORC1/TRIM28 co-occupancy at transposon loci.

We next extracted MORC1-bound transposons from τ-group I, μ-group I, and group II and classified them by transposon family. Each group displayed distinct features: for example, LINE_L1 elements were most abundant in μ-group I, whereas LTR_ERVK elements predominated in τ-group I (Figure 5D). We further quantified the enrichment of individual transposon subfamilies in τ-group I and μ-group I. μ-group I was markedly enriched for evolutionarily young LINE_L1 subfamilies (Figure 5E). Consistently, a separate analysis showed increased TRIM28 occupancy at these young LINE_L1 subfamilies upon MORC1 expression and binding (Figure S5B).

In contrast, τ-group I was biased toward LTR_ERVK elements, with particularly strong enrichment for Intracisternal A-particle (IAP) subfamilies (Figure 5F). Transposons in both μ-group I and τ-group I, but not group II, closely corresponded to those derepressed in MORC1-deficient gonocytes (Figures 5G, S5C, and S5D). μ-group I also showed reduced H3K9me3 in MORC1-deficient gonocytes (Figure 5H), consistent with a link between MORC1 and TRIM28-associated repression.

Together, these results indicate that MORC1-bound transposons can be subdivided into distinct classes based on their relationship to TRIM28, with μ-group I showing the clearest evidence of MORC1-associated TRIM28 recruitment at evolutionarily young LINE_L1 elements.

## Discussion

In this study, we use a Dox-inducible MORC1 expression system in NIH3T3 cells, which do not endogenously express MORC1, to dissect the intrinsic properties of MORC1. This approach revealed two key features of MORC1.

First, MORC1 forms nuclear condensates through an IDR containing a centrally located bpNLS. This property is shared with MORC3 and MORC4, but not with MORC2a or MORC2b. Second, MORC1 strongly interacts with TRIM28 and concentrates it within nuclear condensates. This property appears to be unique to MORC1 among the MORC family members examined.

Our results further suggest that MORC1 condensate formation influences not only the extent of chromatin binding but also the qualitative nature of its binding sites. Whereas WT MORC1 showed limited binding to closed chromatin, ΔIDR was able to occupy closed regions more broadly. These findings suggest that the physical bulk of condensates, or the associated local molecular occupancy, helps shape the chromatin-binding profile of MORC1. In addition, condensate formation may influence the effective quantitative distribution of MORC1 within the nucleus. Consistent with this idea, FRAP analysis showed that condensate formation markedly restricts MORC1 mobility, supporting a model in which nuclear condensates limit the freely diffusible pool of MORC1 available for chromatin exploration.

Although these properties were evident upon ectopic expression in NIH3T3 cells, MORC1 expression did not substantially alter pre-existing TRIM28/SETDB1-dependent repression. One possible explanation is that MORC1 expression in our system was transient. In principle, stable and prolonged MORC1 expression could be established; however, because NIH3T3 cells and other somatic cell types do not normally express MORC1, sustained MORC1 expression may not be well tolerated and could impose a cellular burden. Another possibility is that most MORC1-bound transposons in NIH3T3 cells are already embedded in strongly repressed heterochromatin, limiting the detectable impact of ectopic MORC1 on transcription or chromatin state.

The requirement for MORC1 appears to be highly context-dependent. In gonocytes, MORC1 is essential for transposon silencing, and loss of MORC1 leads to transposon derepression.^3^ Moreover, among MORC family members, MORC1 is the only member highly expressed in gonocytes. These observations suggest that gonocytes represent a cellular context in which the unique properties of MORC1, particularly its strong interaction with TRIM28, are specifically required. MORC1 may, therefore, support efficient transposon silencing by acting together with TRIM28 and SETDB1 at transposon loci.

At the same time, the mechanism by which MORC1 is recruited to specific transposon subfamilies remains unclear. KRAB-ZFPs are plausible candidates, given their established roles in sequence-specific transposon recognition and TRIM28 recruitment, but the involvement of additional factors cannot be excluded. Unlike NIH3T3 cells and other somatic cell types, gonocytes possess the PIWI-piRNA pathway, raising the possibility that this pathway may also contribute, at least in part, to MORC1 targeting or function. In particular, PIWI-piRNA pathway components may act more directly in DNA methylation of young LINE_L1 elements, together with factors such as SPOCD1, SPINDLIN1, and the nuclear pore component TPR.^57^^,^^58^

The role of MORC1 condensates may also differ between NIH3T3 cells and gonocytes. Gonocyte chromatin is thought to be relatively open around the time MORC1 expression begins,^11^ and therefore, the restriction of chromatin binding by bulky condensates may be less pronounced than in NIH3T3 cells. However, as chromatin progressively becomes less accessible, condensate formation may help limit MORC1 access to already closed regions and promote its effective localization to regions that remain accessible. Condensate formation may also fine-tune the local concentration or effective dosage of MORC1-TRIM28 complexes in a manner that cannot be readily achieved by transcriptional or translational control alone.

This model could be tested by expressing MORC3 in MORC1-deficient gonocytes or by expressing an IDR-deleted MORC1 mutant in the same background. Such experiments should help distinguish the contribution of the MORC1-specific TRIM28 interaction from that of condensate formation and thereby clarify how these two properties cooperate to support transposon silencing in gonocytes.

### Limitations of the study

This study primarily relied on a Dox-inducible MORC1 expression system in NIH3T3 fibroblasts. Although this system enabled controlled dissection of MORC1 domains, condensate formation, TRIM28 interaction, and chromatin occupancy, it does not fully recapitulate the gonocyte context, in which endogenous MORC1 acts together with germline-specific pathways and chromatin states. In particular, whether the expression-dependent condensate properties observed in NIH3T3 cells apply to endogenous MORC1 in gonocytes remains unclear, and this is difficult to test directly because of limited cell numbers and developmental heterogeneity.

Although ectopic MORC1 bound transposon loci in NIH3T3 cells, it did not measurably alter transposon expression, likely because these elements are already embedded in repressed heterochromatin. Thus, our data do not directly demonstrate that MORC1-mediated TRIM28 recruitment is sufficient to establish *de novo* transposon silencing.

The mechanism that directs MORC1 to specific transposon subfamilies remains unresolved. KRAB-ZFPs are plausible candidates, but their involvement has not been directly tested. Identifying the upstream targeting factors will be essential for understanding how MORC1 selectively acts on young LINE-1 and other transposon subfamilies in gonocytes.

## Resource availability

### Lead contact

Requests for further information and resources should be directed to and will be fulfilled by the lead contact, Mikiko C. Siomi (siomim@bs.s.u-tokyo.ac.jp).

### Materials availability

All plasmids and cell lines generated in this study are available from the lead contact upon request with a completed materials transfer agreement.

### Data and code availability

• All datasets generated in this study are publicly available as of the date of publication. The accession numbers are listed as follows and in the key resources table.
• All sequence datasets generated in this study have been deposited in the Gene Expression Omnibus (GEO) under the following accession nos.: GSE314168 (RNA-seq from naive NIH3T3 cells), GSE314169 (RNA-seq from NIH3T3 cells expressing 3xFLAG-MORC1 WT or 3xFlag-MORC1 ΔIDR-bpNLS), GSE314166 (MORC1 ChIP-seq from NIH3T3 cells expressing 3xFLAG-MORC1 WT or 3xFlag-MORC1 ΔIDR-bpNLS), GSE314170 (TRIM28 ChIP-seq from NIH3T3 cells expressing 3xFLAG-mCherry, 3xFLAG-MORC1 WT, or 3xFLAG-MORC1 ΔIDR-bpNLS), and GSE314165 (ATAC-seq from NIH3T3 cells expressing 3xFLAG-mCherry, 3xFLAG-MORC1 WT, or 3xFLAG-MORC1 ΔIDR-bpNLS). Detailed information and quality-control metrics for all sequencing datasets generated in this study are provided in Table S2.
• The MS proteomics data have been deposited to the ProteomeXchange Consortium via the jPOST partner repository with the dataset identifier PXD074028.
• This paper does not report original code.
• Any additional information required to reanalyze the data reported in this paper is available from the lead contact upon request.

## Acknowledgments

We thank H. Ishizu for technical advice and experimental consultation and G. Nakayama, D. Nakazato, T. Fujisawa, D. Brenske, N. Yakushiji, Y. Koseki, R. Shimada, and K. Ishiguro for experimental assistance. We also thank H. Koseki, Y. Shinkai, and all members of the Siomi laboratory for discussion. This work was supported by 10.13039/501100001691JSPS KAKENHI grant nos. JP25H01303 (M.C.S.), JP23K14141 (H.Y.), JP24H01271 (H.Y.), JP26KJ0831 (P.L.), and JP25K02253 (S.Y.); 10.13039/501100004298Secom Science and Technology Foundation (H.Y.); JSPS Program for Forming Japan’s Peak Research Universities (J-PEAKS) grant no. JPJS00420240022 (H.K.); 10.13039/100009619Japan Agency for Medical Research and Development grant no. JP21bm0704041 (S.Y.); Medical Research Center Initiative for High Depth Omics (H.K.); and JST SPRING grant no. JPMJSP2108 (P.L.).

## Author contributions

Y.I., H.Y., K.S., H.C., and S.I. performed the biochemical experiments. H.K. performed liquid chromatography-tandem mass spectrometry (LC-MS/MS) analysis. P.L. performed the bioinformatic analyses. S.Y. supervised and discussed the work. H.Y. and M.C.S. supervised and discussed the work and wrote the manuscript with the other authors.

## Declaration of interests

The authors declare no competing interests.

## Declaration of generative AI and AI-assisted technologies in the writing process

During the preparation of this work, the authors used Claude (Anthropic) and ChatGPT (OpenAI) to enhance the clarity and readability of the writing. The authors reviewed and revised all such content and take full responsibility for the final manuscript.

## STAR★Methods

### Key resources table

**Table:** 

REAGENT or RESOURCE	SOURCE	IDENTIFIER
**Antibodies**		

anti-Flag M2 mouse monoclonal	Sigma-Aldrich	Cat# F3165; RRID:AB_259529
anti-β-Tubulin mouse monoclonal	Developmental Studies Hybridoma Bank	Cat# E7; RRID:AB_528499
anti-KAP1 rabbit polyclonal	GeneTex	Cat# GTX102226; RRID:AB_2037324
anti-DDX4 / MVH mouse monoclonal	Abcam	Cat# ab27591; RRID:AB_1113963
anti-MORC1 rabbit polyclonal	Proteintech	Cat# 14080-1-AP; RRID:AB_2266596
anti-mouse IgG (H + L) HRP	Cappel	Cat# 55558; RRID:AB_2334546
anti-rabbit IgG HRP-linked	Cell Signaling Technology	Cat# 7074; RRID:AB_2099233
Alexa Fluor 488 goat anti-mouse IgG1 antibody	Thermo Fisher Scientific	Cat# A-21121; RRID:AB_2535764
Alexa Fluor 555 goat anti-mouse IgG1 antibody	Thermo Fisher Scientific	Cat# A-21127; RRID:AB_2535769
Alexa Fluor 594 goat anti-mouse IgG1 antibody	Thermo Fisher Scientific	Cat# A-21125; RRID:AB_2535767
Alexa Fluor 488 goat anti-rabbit IgG (H + L) antibody	Thermo Fisher Scientific	Cat# A-11008; RRID:AB_143165
Alexa Fluor 555 goat anti-rabbit IgG (H + L) antibody	Thermo Fisher Scientific	Cat# A-21428; RRID:AB_2535849

**Chemicals, peptides, and recombinant proteins**		

DMEM, high glucose	Gibco	Cat# 11965092
Fetal Bovine Serum	Sigma	Cat# 173012
Penicillin-Streptomycin-Glutamine	Gibco	Cat# 10378016
NEBuilder HiFi DNA Assembly Master Mix	New England Biolabs	Cat# E2621X
Ligation high ver.2	TOYOBO	Cat# LGK-201
Lipofectamine 2000	Invitrogen	Cat# 11668019
Opti-MEM I Reduced Serum Medium	Gibco	Cat# 31985062
Dynabeads Protein G	Thermo Fisher Scientific	Cat# 10009D
ClearTrans Nitrocellulose Membrane, 0.2 μm	FUJIFILM Wako Pure Chemical	Cat# 030-25643
Can Get Signal Solution 1 & 2	TOYOBO	Cat# NKB-101
Clarity Western ECL Substrate	Bio-Rad	Cat# 170506
SilverQuest Silver Staining Kit	Thermo Fisher Scientific	Cat# LC6070
VECTASHIELD Antifade Mounting Medium with DAPI	VECTOR LABORATORIES	Cat# H-1200
O.C.T. Compound	Sakura Finetek Japan	Cat# 45833
Hoechst 33342	Lonza	Cat# PA-3014
ISOGEN II	NIPPON GENE	Cat# 311-07361
TURBO DNase	Thermo Fisher Scientific	Cat# AM2239
TruSeq stranded mRNA	Illumina	Cat# RS-122-2103
NEBNext Ultra II Directional RNA Library Prep Kit	New England Biolabs	Cat# E7765L
truChIP Chromatin Shearing Kit with Formaldehyde	Covaris	Cat# 520154
Proteinase K	Roche	Cat# 03115828001
NEBNext Ultra Ⅱ DNA Library Prep Kit for Illumina	New England Biolabs	Cat# E7645S
Nextera DNA Library Prep Kit	Illumina	Cat# FC-121-1030
MinElute PCR Purification Kit	Qiagen	Cat# 28004
Trypsin/Lys-C Mix	Promega	Cat# V5072

**Critical commercial assays**		

ChemiDoc XRS Plus System	Bio-Rad	Cat# 1708265J1PC
Stage Top Incubator	TOKAI HIT	Cat# STXG-WSKMX-SET
Branson digital sonifier 250D-Advanced	BRANSON	Cat# 101-063-838
Branson digital sonifier SFX150	BRANSON	Cat# P1CTK1000019-1
GL-Tip SDB	GL Sciences	Cat# 7820-11200
Nanoelectrospray ion source	Thermo Fisher Scientific	Cat# IQLAAEGABBFADNMAIF
75 μm inner diameter × 150 mm C18 reversed-phase column	Nikkyo Technos	N/A

**Deposited data**		

Raw sequencing data	This paper	GEO: GSE314165 GEO: GSE314166 GEO: GSE314168 GEO: GSE314169 GEO: GSE314170
Raw mass spectrometry proteomics data	This paper	jPOST: PXD074028
Adult germ cell RNA-seq data	Hasegawa et al.^36^ Maezawa et al.^37^	GEO: GSE55060 GEO: GSE89502
*Morc1* KO and Het Gonocyte RNA-seq data	Uneme et al.^11^	GEO: GSE235429
*Morc1* KO and Het Gonocyte ChIP-seq data	Uneme et al.^11^	GEO: GSE235429

**Experimental models: Cell lines**		

NIH3T3	RIKEN BioResource Research Center	Cat# RCB2767, RRID:CVCL_0594

**Experimental models: Organisms/strains**		

Ddx4-Venus TG Mouse	Ogonuki et al.^59^	N/A
C57BL/6J	Japan SLC	C57BL/6JJmsSlc

**Recombinant DNA**		

Caspex	Myers et al.^60^	Cat# 97421, RRID:Addgene_97421
pEGFPC1	Abrisch et al.^61^	Cat# 141152, RRID:Addgene_141152
pCMV-hyPBase	Yusa et al.^62^	N/A
Caspex-3xFlag-mCherry	This study	N/A
Caspex-3xFlag-Morc1 WT	This study	N/A
Caspex-3xFlag-Morc1 ΔIDR-bpNLS	This study	N/A
pEGFPC1-3xFlag-Morc1 WT	This study	N/A
pEGFPC1-3xFlag-Morc1 ΔIDR	This study	N/A
pEGFPC1-3xFlag-Morc1 bpNLS_m1	This study	N/A
pEGFPC1-3xFlag-Morc1 bpNLS_m2	This study	N/A
pEGFPC1-3xFlag-Morc1 ΔIDR-bpNLS	This study	N/A
pEGFPC1-3xFlag-mCherry-MVH	This study	N/A
pEGFPC1-3xFlag-NLS-mCherry-MVH	This study	N/A
pEGFPC1-mCherry-Trim28 WT	This study	N/A
pEGFPC1-mCherry-Trim28 ΔPHD	This study	N/A
pEGFPC1-mCherry-Trim28 ΔBROMO	This study	N/A
pEGFPC1-mCherry-Trim28 ΔNLS	This study	N/A
pEGFPC1-mCherry-Trim28 ΔNLSΔBROMO	This study	N/A
pEGFPC1-3xFlag-Morc2a	This study	N/A
pEGFPC1-3xFlag-Morc2b	This study	N/A
pEGFPC1-3xFlag-Morc3	This study	N/A
pEGFPC1-3xFlag-Morc4	This study	N/A
pEGFPC1-3xFlag-Morc1 3-IDR	This study	N/A
pEGFPC1-3xFlag-Morc1 3-CW/ IDR	This study	N/A

**Software and algorithms**		

STRING (Ver. 12.0)	Szklarczyk et al.^63^	RRID:SCR_005223; https://string-db.org/
DISOPRED3	Jones and Cozzetto^40^	RRID:SCR_010248; https://bioinf.cs.ucl.ac.uk/psipred/
cNLS Mapper	Kosugi et al.^41^^,^^42^^,^^43^	https://nls-mapper.iab.keio.ac.jp/cgi-bin/NLS_Mapper_form.cgi
CLUSTAL Omega (version 1.2.4)	Sievers et al.^64^	RRID:SCR_001591 ; https://www.ebi.ac.uk/jdispatcher/msa/clustalo
Fiji (ImageJ, version 2.1.0/1.53c)	Schindelin et al.^65^	RRID:SCR_002285 ; https://fiji.sc
Proteome Discoverer 2.5	Thermo Fisher Scientific	RRID: SCR_014477
Cutadapt	Kechin et al.^66^	RRID: SCR_011841; https://cutadapt.readthedocs.io/en/stable/
SeqKit	Shen et al.^67^	RRID: SCR_018926; https://bioinf.shenwei.me/seqkit/
FastQC	N/A	RRID:SCR_014583; https://www.bioinformatics.babraham.ac.uk/projects/fastqc/
Trim Galore	N/A	RRID:SCR_011847; http://www.bioinformatics.babraham.ac.uk/projects/trim_galore/
Bowtie2	Langmead and Salzberg^68^	RRID: SCR_016368; https://bowtie-bio.sourceforge.net/bowtie2/index.shtml
STAR	Dobin et al.^69^	RRID:SCR_004463; https://github.com/alexdobin/STAR
featureCounts	Liao et al.^70^	RRID:SCR_012919; https://subread.sourceforge.net/featureCounts.html
Picard	N/A	RRID:SCR_006525; http://broadinstitute.github.io/picard/
Samtools	Danecek et al.^71^	RRID:SCR_002105; http://htslib.org/
bamCoverage	Ramírez et al.^72^	RRID:SCR_016366; https://deeptools.readthedocs.io/en/develop/
MACS3	Zhang et al.^73^	RRID:SCR_013291; https://macs3-project.github.io/MACS/
DESeq2	Love et al.^74^	RRID:SCR_015687; https://bioconductor.org/packages/release/bioc/html/DESeq2.html
edgeR	Chen et al.^75^	RRID:SCR_012802; https://bioconductor.org/packages/release/bioc/html/edgeR.html
BEDTools	Quinlan and Hall^76^	RRID:SCR_006646; https://github.com/arq5x/bedtools2
GenMap	Sedlazeck et al.^77^	RRID:SCR_005488; http://cibiv.github.io/NextGenMap/
deepTools	Ramírez et al.^72^	RRID:SCR_016366; https://deeptools.readthedocs.io/en/develop/
ggplot2	https://link.springer.com/book/10.1007/978-3-319-24277-4	RRID:SCR_014601; https://ggplot2.tidyverse.org/
clusterProfiler	Yu et al.^78^	RRID:SCR_016884; https://bioconductor.org/packages/release/bioc/html/clusterProfiler.html

### Experimental model and study participant details

#### Mice

Wild-type C57BL/6JmsSlc mice were purchased from Japan SLC Inc. and maintained under standard laboratory conditions at The University of Tokyo. All animal procedures were approved by the Institutional Safety Committee on Recombinant DNA Experiments and the Animal Research Committee of the Graduate School of Science, The University of Tokyo (approval no. A2025S005-02), and were performed in accordance with the guidelines for animal experiments at The University of Tokyo. For timed mating, the day on which a vaginal plug was detected was designated as embryonic day 0.5 (E0.5). Embryos were collected at E17.5, and only testes from male embryos were used for subsequent analyses.

This study focused on the male germline, in which MORC1 functions in gonocytes. Therefore, only male embryos were analyzed. As female germ cells were not examined, the potential influence of sex on the reported findings could not be assessed and represents a limitation of this study.

#### Cell lines

NIH3T3 cells (RRID:CVCL_0594) were obtained from the RIKEN BioResource Research Center (Cat# RCB2767). Doxycycline (Dox)-inducible stable cell lines were generated from these cells in this study. NIH3T3 cells were cultured at 37°C under 5% CO_2_ in Dulbecco's Modified Eagle Medium (Gibco) supplemented with 10% fetal bovine serum (Sigma) and Penicillin-Streptomycin-Glutamine (Gibco). The cells were not independently authenticated in this study but were obtained directly from the RIKEN BioResource Research Center. The cells tested negative for mycoplasma contamination.

### Method details

#### Plasmid construction

Coding sequences for mouse MORC1, MORC2a, MORC2b, MORC3, MORC4, and MVH were amplified by PCR from either mouse testis cDNA or NIH3T3 cDNA library, depending on the gene. The TRIM28 open reading frame was obtained by gene synthesis (Invitrogen GeneArt). PCR products and linearized vector fragments were assembled into either pEGFPC1^61^ or Caspex^60^ backbone by NEBuilder HiFi DNA Assembly Master Mix (New England Biolabs). This strategy was used to generate the MORC1 WT construct, MORC1 deletion derivatives, the MORC1 3-IDR and 3-CW/IDR variants, MORC paralog constructs, and the mCherry- or Flag-tagged MVH and TRIM28 WT expression plasmids. Site-directed mutagenesis for MORC1 bpNLS variants and TRIM28 domain-deletion constructs was performed by inverse PCR followed by Ligation high ver.2 (TOYOBO). Details of the PCR primers and plasmid constructs used in this study are provided in Table S3.

#### Plasmid transfection

NIH3T3 cells were seeded one day before to reach ∼70–90% confluency at the time of transfection. Plasmid DNA was diluted in Opti-MEM I Reduced Serum Medium (Gibco) and transfected using Lipofectamine 2000 (Thermo Fisher Scientific) according to the manufacturer’s instructions. For co-transfections, plasmids were mixed at equimolar ratios while keeping the total DNA amount constant. Medium was not exchanged after transfection. Cells were harvested or processed 48 h post-transfection for downstream analyses.

#### Generation of Dox-inducible stable cell lines

Dox-inducible NIH3T3 cell lines expressing 3xFlag-mCherry, 3xFlag-MORC1 WT, or 3xFlag-MORC1 ΔIDR-bpNLS were generated using the piggyBac transposon system. Donor plasmids (Caspex-3xFlag-mCherry, Caspex-3xFlag-MORC1 WT, or Caspex-3xFlag-MORC1 ΔIDR-bpNLS) and the piggyBac transposase expression vector pCMV-hyPBase^62^ were co-transfected at a 5:1 (w/w) ratio. At 48 h post-transfection, cells were reseeded at approximately 1/21 of the original density. The following day, puromycin was added to the medium at 2 μg/mL for selection; medium was replaced every 3 days. Surviving colonies were picked individually and sequentially expanded from 24-well plates to 35 mm, 6 cm, and 10 cm dishes. Transgene expression was induced by addition of Dox (100 ng/mL) and cells were used for experiments 48 h after Dox addition.

#### Co-immunoprecipitation

Cells were harvested by centrifugation and resuspended in hypotonic buffer [20 mM HEPES-KOH (pH 7.3), 10 mM KCl, 1.5 mM MgCl_2_, 0.5 mM DTT, 2 μg/mL pepstatin A, 2 μg/mL leupeptin, and 0.5 μg/mL aprotinin]. The suspension was pipetted and passed through a 25-gauge needle three times, then centrifuged; the pellet was washed three times with hypotonic buffer. The pellet was resuspended in co-IP buffer [50 mM HEPES-KOH (pH 7.3), 200 mM KCl, 1 mM EDTA, 1% Triton X-100, 0.1% sodium deoxycholate, 2 μg/mL pepstatin A, 2 μg/mL leupeptin, and 0.5 μg/mL aprotinin] and sonicated (Branson digital sonifier 250D-Advanced; amplitude 20%, 0.2 s ON / 0.8 s OFF cycles, total ON-time 1 min). Lysates were clarified by centrifugation at ∼20,000 × g for 20 min at 4°C. Dynabeads Protein G (Thermo Fisher Scientific) were washed with co-IP buffer and incubated with Flag M2 antibody (Sigma-Aldrich) for ≥30 min at room temperature with rotation to allow antibody binding. Clarified lysate was split and an input fraction reserved; the remainder was incubated with the antibody-bound beads for 2 h at 4°C with rotation. Beads were washed five times with co-IP buffer. Bound proteins were eluted in 2× SDS sample buffer [125 mM Tris-HCl (pH 6.8), 4% SDS, 19% glycerol, 200 mM DTT, and 0.01% bromophenol blue] at 70°C for 10 min prior to SDS-PAGE analysis.

#### Western blotting and silver staining

Protein samples denatured in the sample buffer and boiled at 95°C for 5 min. The proteins were separated by sodium dodecyl sulfate polyacrylamide electrophoresis using 8% polyacrylamide gels. After electrophoresis, they were transferred to ClearTrans Nitrocellulose Membrane (FUJIFILM Wako Pure Chemical). The membranes were blocked with 5% skim milk/PBS for 30 min at room temperature, primary antibodies diluted in Can Get Signal Solution 1 (TOYOBO) were applied overnight at 4°C. After washing, membranes were incubated with HRP-conjugated secondary antibodies diluted in Can Get Signal Solution 2 (TOYOBO) for 30 min at room temperature and signals detected using Clarity Western ECL Substrate (Bio-Rad) and imaged on ChemiDoc XRS Plus System (Bio-Rad). The following primary antibodies were used: anti-Flag (Sigma-Aldrich, 1:5,000 dilution), anti-β-Tubulin (Developmental Studies Hybridoma Bank, 1:1,000 dilution), anti-KAP1 (GeneTex, 1:1,000 dilution). The following secondary antibodies were used: anti-mouse IgG (H + L) HRP (Cappel, 1:5,000 dilution), anti-rabbit IgG HRP-linked (Cell Signaling Technology, 1:1,000 dilution). Silver staining was performed using SilverQuest Silver Staining Kit (Thermo Fisher Scientific) following the manufacturer’s instructions.

#### Immunofluorescence of NIH3T3 cells

NIH3T3 cells were seeded on sterile glass coverslips placed in culture wells prior to transfection. At 48 h post-transfection, cells were fixed with methanol at room temperature for 5 min, followed by three washes with PBS containing 0.1% Tween-20 (PBS-T) for 10 min each. Cells were blocked with 1% BSA in PBS for 1 h at room temperature and incubated with primary antibodies diluted in 1% BSA/PBS for 1 h at room temperature. After three washes with PBS-T for 10 min each, cells were incubated with Alexa Fluor-conjugated secondary antibodies diluted in 1% BSA/PBS for 1 h at room temperature in the dark. Samples were washed three times with PBS-T and mounted with VECTASHIELD Antifade Mounting Medium with DAPI (VECTOR LABORATORIES). The following primary antibodies were used: anti-Flag (Sigma-Aldrich, 1:3,000 dilution), anti-KAP1 (GeneTex, 1:1,000 dilution). The following secondary antibodies were used: Alexa Fluor 488/555/594 goat anti-mouse IgG1 (Thermo Fisher Scientific, 1:1,000 dilution), Alexa Fluor 488 goat anti-rabbit IgG (H + L) (Thermo Fisher Scientific, 1:1,000 dilution).

#### Immunofluorescence of testes

Mouse testes were fixed in 4% paraformaldehyde (pH 7.0) on ice for 1 h, washed four times with PBS, and cryoprotected sequentially in 10%, 20% and 30% sucrose in PBS at 4°C until tissues sank. Testes were embedded in O.C.T. Compound (Sakura Finetek Japan) and stored at −80°C. Cryosections (10 μm) were prepared and mounted onto glass slides. Sections were washed with Milli-Q water, subjected to antigen retrieval in 0.01 M citrate buffer (citric acid and trisodium citrate at a molar ratio of 1:4.6) at 105°C for 15 min, blocked with 3% skim milk in PBS-T for 30 min, and incubated with primary antibodies diluted in 3% skim milk/PBS-T for 1 h at room temperature. After three washes with PBS-T, sections were incubated with Alexa Fluor-conjugated secondary antibodies diluted in 3% skim milk/PBS-T for 1 h at room temperature in the dark. Slides were washed and mounted with VECTASHIELD Antifade Mounting Medium with DAPI (VECTOR LABORATORIES). The following primary antibodies were used: anti-MORC1 (Proteintech, 1:1,000 dilution), anti-MVH/DDX4 (Abcam 1:1,000 dilution). The following secondary antibodies were used: Alexa Fluor 488 goat anti-mouse IgG1 (Thermo Fisher Scientific, 1:1,000 dilution), Alexa Fluor 555 goat anti-rabbit IgG (H + L) (Thermo Fisher Scientific, 1:1,000 dilution).

#### Live-cell imaging

NIH3T3 cells were cultured in glass-bottom dishes and transfected as described above. At 48 h post-transfection, nuclei were stained with Hoechst 33342 (Lonza) according to the manufacturer’s instructions. Live-cell imaging was performed using Stage Top Incubator (TOKAI HIT) to maintain cells at 37°C and 5% CO_2_ during image acquisition. For the FRAP assay, the fluorescent signal of mCherry was bleached using 561-nm laser light and observed by time-lapse imaging.

#### RNA extraction and RNA-seq library construction

Total RNA was isolated from NIH3T3 cells using ISOGEN II (NIPPON GENE) according to the manufacturer’s protocol. To remove residual genomic DNA, purified RNA was treated with Turbo DNase (Thermo Fisher Scientific) at 37°C for 30 min. DNase-treated RNA was then purified by phenol–chloroform extraction, followed by chloroform cleanup. The aqueous phase was subjected to ethanol precipitation with sodium acetate, washed with 70% ethanol, air-dried, and dissolved in nuclease-free water. RNA-seq libraries of wild-type NIH3T3 cells were prepared using TruSeq stranded mRNA (illumina) following manufacturer’s protocols and sequenced on an Illumina NovaSeq 6000 platform. RNA-seq libraries of NIH3T3 cells expressing 3xFlag-mCherry, 3xFlag-MORC1 WT, or 3xFlag-MORC1 ΔIDR-bpNLS were prepared using NEBNext Ultra II Directional RNA Library Prep Kit (New England Biolabs) following manufacturer’s protocols and sequenced on an Illumina NovaSeq X Plus platform. Both RNA-seq datasets include three biological replicates for each condition.

#### ChIP-seq library construction

Dox-inducible NIH3T3 cell lines expressing 3xFlag-mCherry, 3xFlag-MORC1 WT, or 3xFlag-MORC1 ΔIDR-bpNLS were treated with Dox for 24 h prior to the experiment, and 1 × 10^6^ cells were used for each ChIP-seq sample. Two biological replicates were included for each condition. Cells were fixed with 1% formaldehyde for 20 min and resuspended in Swelling buffer [20 mM Hepes (pH 7.9), 1.5 mM MgCl2, 10 mM KCl, 0.1% NP-40, and 1 mM DTT]. After incubation on ice for 20 min, pelleted nuclei were resuspended in 1× shearing buffer (Covaris) and fragmented to ∼500 bp with Branson digital sonifier SFX150 (BRANSON). Fragmented products diluted with RIPA buffer [50 mM Tris-HCl (pH 8.0), 150 mM NaCl, 2 mM EDTA (pH 8.0), 1% NP-40, 0.5% sodium deoxycholate, and 0.1% SDS] were mixed by rotation with Dynabeads Protein G (Thermo Fisher Scientific) for 1 h at 4°C. Immunoprecipitation was performed with 2 μL anti-Flag (Sigma-Aldrich), or 4 μL anti-KAP1 (GeneTex) on Dynabeads overnight at 4°C. Beads were washed with Low buffer [0.1% SDS, 1% Triton X-100, 2 mM EDTA (pH 8.0), 150 mM NaCl, and 20 mM Tris-HCl (pH 8.0)] three times and then with High buffer [0.1% SDS, 1% Triton X-100, 2 mM EDTA (pH 8.0), 500 mM NaCl, and 20 mM Tris-HCl (pH 8.0)] once. Immunoprecipitated products were eluted from beads by suspension in Direct elution buffer [10 mM Tris-HCl (pH 8.0), 5 mM EDTA (pH 8.0), 300 mM NaCl, and 0.5% SDS] while shaking at 65°C for 15 min. The sample was treated with Proteinase K for 6 h at 37°C, followed by reversal of cross-linking overnight at 65°C. DNA was extracted by ethanol precipitation. Library preparation was carried out with a NEBNext Ultra Ⅱ DNA Library Prep Kit for Illumina (NEB) following the manufacturer’s instructions.

#### ATAC-seq library construction

ATAC-seq was carried out following established protocols,^79^^,^^80^ with minor adjustments for this study. Dox-inducible NIH3T3 cells expressing 3×Flag-mCherry, 3×Flag-MORC1 (WT), or 3×Flag-MORC1 ΔIDR-bpNLS were collected after 48 h of Dox induction (1 × 10^5^ cells per reaction). Two biological replicates were included for each condition. Cells were gently lysed in 50 μL of ice-cold lysis buffer to release nuclei, which were subsequently pelleted and transferred into a transposition mixture composed of 2× TD buffer (25 μL; Illumina), TDE1 transposase (1.5 μL; Illumina), and nuclease-free water (23.5 μL). The transposition reaction proceeded at 37°C for 30 min. DNA fragments generated by TDE1 insertion were then purified using MinElute PCR Purification Kit (Qiagen). Libraries were produced by PCR amplification using barcoded primer sets (0.125 μM). Transposition performance and library quality were evaluated by TapeStation analysis of fragment size distribution prior to sequencing.

#### Computational and image analysis

For the analysis of protein interactions, proteins demonstrating significant binding to MORC1 WT, in comparison to the control (309 proteins, log2Fold Change > 1 and P-value < 0.05), as well as SETDB1 (UniProt accession ID: O88974), were submitted to STRING (Version 12.0).^63^ Subsequently, proteins interacting with SETDB1 were identified and extracted. The IDRs of MORC1 were predicted using DISOPRED3^40^ via the PSIPRED server. Nuclear localization signals (NLSs) were predicted using cNLS Mapper^41^^,^^42^^,^^43^ with a cut-off score of 5.0. Multiple sequence alignment of mouse MORC1–4 amino acid sequences was performed using CLUSTAL Omega (version 1.2.4)^64^ with default parameters. A phylogenetic tree was generated from the alignment using the Neighbor-Joining method provided by CLUSTAL Omega. For nuclear foci analysis, images of NIH3T3 cells and gonocytes expressing MORC1 WT were processed in Fiji (ImageJ, version 2.1.0/1.53c).^65^ Images were binarized using the Max Entropy thresholding method, and nuclear foci were detected using the Analyze Particles function to measure the area of each focus. To exclude noise, objects with an area of 0.017 μm^2^ or less were removed from subsequent analyses. For FRAP analysis, the fluorescence intensity of the bleached region of interest was quantified at each time point using Fiji. The values were normalized such that the mean intensity of the two pre-bleach frames was standardized to 1, while the intensity immediately following the bleaching process was standardized to 0. Subsequently, the normalized recovery curves were fitted to the equation I(t) = Imax × (1 − √ (τ / (τ + π t))) + C using the curve_fit function in SciPy (Python). For co-immunoprecipitation analysis, the signal intensity of each band was quantified using Fiji, and the background was subtracted.

#### LC–MS/MS analysis

Proteins bound to the beads were digested with 200 ng of trypsin/Lys-C mix (Promega) at 37°C overnight. The resulting peptides were reduced, alkylated, acidified, and desalted using GL-Tip SDB (GL Sciences). Eluates were dried in a SpeedVac concentrator and reconstituted in 0.1% trifluoroacetic acid and 3% acetonitrile (ACN). LC-MS/MS analysis was performed on an EASY-nLC 1200 UHPLC system coupled to an Orbitrap Fusion mass spectrometer equipped with a nanoelectrospray ion source (Thermo Fisher Scientific). The peptides were separated on a 75 μm inner diameter × 150 mm C18 reversed-phase column (Nikkyo Technos) with a linear 4–32% ACN gradient for 0–100 min followed by an increase to 80% ACN for 10 min and a final hold at 80% ACN for 10 min. The mass spectrometer was operated in a data-dependent acquisition mode with a maximum duty cycle of 3 s. MS1 spectra were acquired with a resolution of 120,000, an automatic gain control (AGC) target of 4e5, and a mass range from 375 to 1,500 m/z. HCD MS/MS spectra were acquired in the linear ion trap with an AGC target of 1e4, an isolation window of 1.6 m/z, a maximum injection time of 35 ms, and a normalized collision energy of 30. Dynamic exclusion was set to 20 s. Raw data were searched against SwissProt database restricted to *Mus musculus* using Proteome Discoverer version 2.5 (Thermo Fisher Scientific, USA) with the Sequest HT search engine. The search parameters were as follows: (a) trypsin as an enzyme with up to two missed cleavages; (b) precursor mass tolerance of 10 ppm; (c) fragment mass tolerance of 0.6 Da; (d) carbamidomethylation of cysteine as a fixed modification; and (e) acetylation of protein N-terminus and oxidation of methionine as variable modifications. Peptides were filtered at a false discovery rate of 1% using the Percolator node. Label-free precursor ion quantification was performed using the Precursor Ions Quantifier node, and normalization was performed such that the total sum of abundance values for each sample over all peptides was the same. The raw LC-MS/MS data are provided in Table S1.

#### RNA-seq analysis

RNA-seq data from WT NIH3T3 cells were mapped to the mouse genome (mm10) using Bowtie2 in paired-end mode after quality check. Uniquely mapped reads were retained, while unmapped reads and reads overlapping genomic blacklist regions (mm10-blacklist.v2) were removed. Read counts on gene exons were then calculated using featureCounts with the options -p -t exon -g gene_id. RNA-seq data from Dox-inducible NIH3T3 cells expressing 3×Flag-mCherry, 3×Flag-MORC1 WT, or 3×Flag-MORC1 ΔIDR-bpNLS were first adapter-trimmed using TrimGalore. For gene expression analysis, trimmed reads were processed using the same pipeline as that used for WT NIH3T3 cells. Gonocyte RNA-seq data was obtained from the Gene Expression Omnibus (GEO) under accession number GSE235429.^11^ Reads were aligned to mouse genome (mm10) using Hisat2 with paired-end mode and --dta option after removing adaptors using Cutadapt and being trimmed to 90bp using SeqKit. Adult germ cell RNA-seq data was obtained from the Gene Expression Omnibus (GEO) under accession number GSE55060,^36^ and GSE89502.^37^ Reads were aligned to mouse genome (mm10) using Bowtie2 after removing adaptors using Cutadapt and being trimmed to 25bp using SeqKit. Reads aligned to genomic blacklist regions (mm10-blacklist.v2) were removed and read counts on gene exons were calculated using featureCounts with -p -t exon -g gene_id options. Transcripts per million (TPM) were calculated for each gene to measure expression level. For transposon expression analysis, subsequent procedures were conducted according to the pipeline described in a previous study.^81^ Specifically, trimmed reads were mapped to mouse genome (mm10) using STAR with following parameters --runMode alignReads --outFilterMultimapNmax 5000 --outSAMmultNmax 1 --outFilterMismatchNmax 3 -outMultimapperOrder Random --winAnchorMultimapNmax 5000 --alignEndsType EndToEnd --alignIntronMax 1 --alignMatesGapMax 350 --seedSearchStartLmax 30 --alignTranscriptsPerReadNmax 30000 --alignWindowsPerReadNmax 30000 --alignTranscriptsPerWindowNmax 300 --seedPerReadNmax 3000 --seedPerWindowNmax 300 --seedNoneLociPerWindow 1000. Read counts for transposons were summarized at the subfamily level based on the RepeatMasker (open-4.0.5) annotation using featureCounts, with the -p option to count paired-end fragments and the -M option to include multi-mapping reads. Differentially expressed transposons between two conditions were identified using DESeq2 based on replicate count matrices after adding pseudocounts. Size-factor normalization was performed using the default median-ratio method, and differential expression was tested using the Wald test implemented in DESeq2. Transposons after removing entries annotated as Simple_repeat, Low_complexity, Satellite, rRNA, snRNA, tRNA, scRNA and other non-transposon categories were used for subsequent analysis. RNA-seq data of *Morc1* KO and Het gonocytes was obtained from the Gene Expression Omnibus (GEO) under accession number GSE235429,^11^ and the same pipeline described above was used for transposon expression analysis.

#### ChIP-seq and ATAC-seq analysis

Adaptors of ChIP-seq reads were removed using Cutadapt, and reads were trimmed to 75 bp using SeqKit. Trimmed reads were aligned to the mouse genome (mm10) using Bowtie2 in paired-end mode. ATAC-seq reads were processed in the same way, with adaptor removal by Cutadapt followed by mapping to mm10 using Bowtie2 in paired-end mode with the options -N 1 -X 2000 --no-mixed --no-discordant. For aligned reads from both ChIP-seq and ATAC-seq, duplicates were removed using Picard, and only HighSignal regions within genomic blacklist regions (mm10-blacklist.v2) were removed. Read and library quality were assessed using Picard CollectInsertSizeMetrics for both ChIP-seq and ATAC-seq datasets. In addition, ATAC-seq-specific quality control was performed using ATACseqQC. The corresponding quality control metrics are summarized in Table S2. Replicate correlations were assessed on filtered transposon regions using the multiBamSummary BED-file function in deepTools, and on customized 50-kb genomic bins using mapped read counts calculated with the BEDTools multicov function. Then, BAM files from biological replicates were merged, and bigWig files were generated using the deepTools function bamCoverage with CPM normalization. Peaks on the merged BAM files were identified using MACS3 with the parameters -f BAMPE --nolambda --keep-dup all. Enriched peaks for each sample were defined using the filterByExpr function in edgeR, with a minimum total count threshold of 10 for ChIP-seq peaks and 50 for ATAC-seq peaks. 21,846 protein-coding genes on autosomes and sex chromosomes were identified based on Mus_musculus.GRCm38.102.gtf from Ensemble (https://asia.ensembl.org/Mus_musculus/Info/Index) for quantitative analysis. Promoters were defined as ± 500 bp around the transcription start site (TSS). The mm10 transposon reference annotation was obtained from RepeatMasker (open-4.0.5), and entries annotated as Simple_repeat, Low_complexity, Satellite, rRNA, snRNA, tRNA, scRNA and other non-transposon categories were removed. Overlaps between gene promoters or transposons and peaks were identified using the intersect function in BEDTools. Genome-wide mappability was calculated with GenMap using the options -K 30 -E 2 -t -w, and the mean mappability for each transposon locus was obtained by intersecting the GenMap bedGraph with transposon coordinates using the map function in BEDTools. H3K9me3 and H3 ChIP-seq datasets were obtained from the Gene Expression Omnibus (GEO) under accession number GSE235429,^11^ and analyzed as previously described.^11^ ChIP-seq reads were normalized to CPM, and relative H3K9me3 enrichment was calculated by dividing the H3K9me3 ChIP signal by the corresponding H3 ChIP signal. The resulting normalized H3K9me3 bigWig files were used for visualization.

### Quantification and statistical analysis

Quantification of microscopy images, including the area of MORC1 nuclear foci and fluorescence recovery in FRAP experiments, was performed using Fiji. All statistical analyses were performed in Python. The statistical test used, the number of independent samples or measurements (n), and the definition of any error bars are indicated in the corresponding figure legends. Two-group comparisons of non-normally distributed data, including MORC1 foci area and the FRAP recovery time constant (τ), were assessed using the two-sided Mann–Whitney U test. Reproducibility between biological replicates of ChIP-seq, ATAC-seq, and RNA-seq, as well as comparisons of read counts between samples, was evaluated using Spearman's rank correlation coefficient. The statistical significance of overlaps between the indicated feature sets, including transposons or promoters bound by MORC1 WT and MORC1 ΔIDR, was assessed using a one-sided Fisher's exact test. Differential expression of transposon subfamilies was analyzed using DESeq2. *p* values were adjusted for multiple testing using the Benjamini–Hochberg method, and significance was determined based on the FDR thresholds indicated in the corresponding figures. In box plots, the box boundaries indicate the 25th and 75th percentiles, the line within the box indicates the median, the whiskers indicate the minimum and maximum values, and circles indicate outliers. For ChIP-seq, ATAC-seq, and RNA-seq, two biological replicates were analyzed per condition, and FRAP recovery was measured from 11 independent measurements per construct. Statistical significance is denoted in the figures as follows: ∗∗∗*p* < 0.001. Where exact *p* values are provided, they are shown above the corresponding plots.

## Supplemental information

Document S1. Figures S1–S5

Table S1. Raw LC-MS/MS data

Table S2. Detailed information and quality-control metrics for all sequencing datasets

Table S3. PCR primers and plasmid constructs
