# Supplementary material for: MORC1 coordinates TRIM28 interaction and nuclear condensate formation for transposon silencing
Source: iScience. 2026 Aug 31;29(9):117394. doi: 10.1016/j.isci.2026.117394 (PMC13562400; doi:10.1016/j.isci.2026.117394)
Supplement: Supplementary file 1 [file mmc1.pdf]

iScience, Volume *29*

Supplemental information

MORC1 coordinates TRIM28 interaction and nuclear condensate formation for transposon silencing

Yosuke Isota, Peilin Li, Kiichiro Sakata, Hiyu Chiwata, Hidetaka Kosako, Soichiro Yamanaka, Shinsuke Ito, Hiroya Yamazaki, and Mikiko C. Siomi

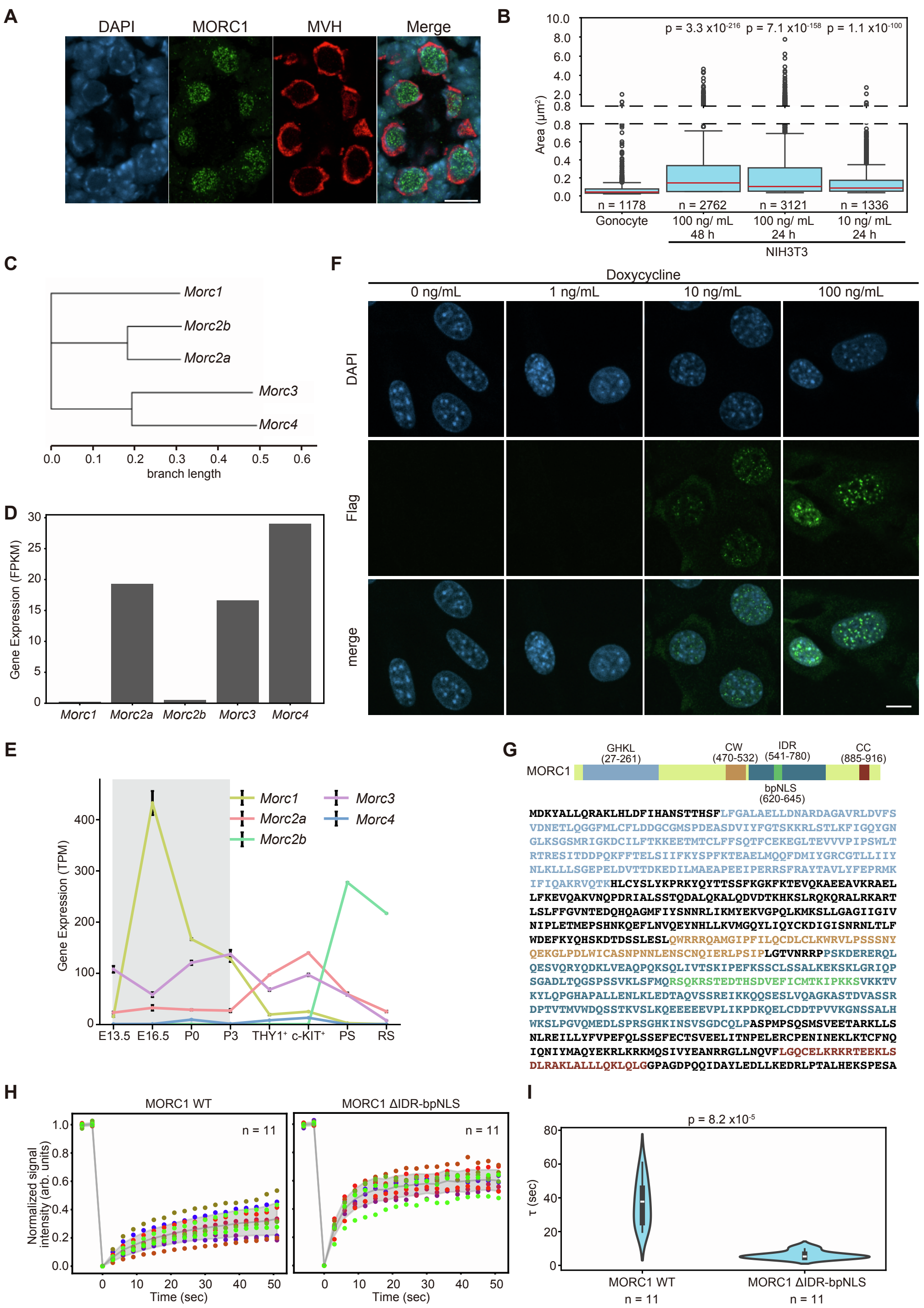

Figure S1. Mouse MORC family members.

(A) Endogenous MORC1 (green) forms discrete nuclear foci ("MORC1 bodies") in MVH-positive (red) gonocytes in the E17.5 fetal testis. Scale bar, 10 μm. **(B)** Box plots showing the distribution of MORC1 nuclear foci area across four conditions: gonocytes , NIH3T3

cells induced with 100 ng/mL Dox for 48 h, 100 ng/mL Dox for 24 h, and 10 ng/mL Dox for 24 h. The box plot represents the minimum and maximum values with whiskers, the 25th and 75th percentiles with the boundaries of the box, the median with a red line inside the box, and the outliers with circles. P values from Mann–Whitney U tests comparing each NIH3T3 cells condition to gonocytes are shown above the respective plots. **(C)** Phylogenetic tree of mouse *Morc* family members (*Morc1–4*). Branch lengths represent the number of substitutions per site. **(D)** Bar plots showing the expression levels of *Morc* family members in NIH3T3 cells. **(E)** Line plots showing the expression levels of *Morc* family members during mouse male germ cell development, at embryonic stages E13.5 to P3 and in postnatal germ cell populations: THY1$^+$ spermatogonia (undifferentiated SSCs), c-KIT$^+$ spermatogonia (differentiating SSCs), pachytene spermatocytes (PS), and round spermatids (RS). Lines show the mean, and error bars indicate the standard deviation. **(F)** Representative fluorescence images of NIH3T3 cells induced with 0, 1, 10, or 100 ng/mL Dox for 24 h, showing dose-dependent expression of Flag-MORC1 (green) and nuclear condensate formation. Nuclei (white dotted outline) stained with DAPI (blue). Scale bar, 10 μm. **(G)** MORC1 peptide sequence and domains. GHKL (gray, aa 27–261); CW (orange, aa 470–532); IDR (blue, aa 541–780); bpNLS (green, aa 620–645); CC (brown, aa 885–916). **(H)** FRAP analysis of MORC1 WT (left) and MORC1 ΔIDR-bpNLS (right). Gray lines indicate mean normalized fluorescence recovery, and gray shading indicates standard deviation. Individual recovery curves from 11 independent measurements are overlaid as dot plots, with each measurement shown in a distinct color. **(I)** Violin plots showing the distribution of the FRAP recovery time constant (τ) for MORC1 WT and ΔIDR-bpNLS. White horizontal bars indicate medians. The box plot represents the minimum and maximum values with whiskers and

the 25th and 75th percentiles with the boundaries of the box. The P value from a Mann–Whitney U test is shown above the plot.

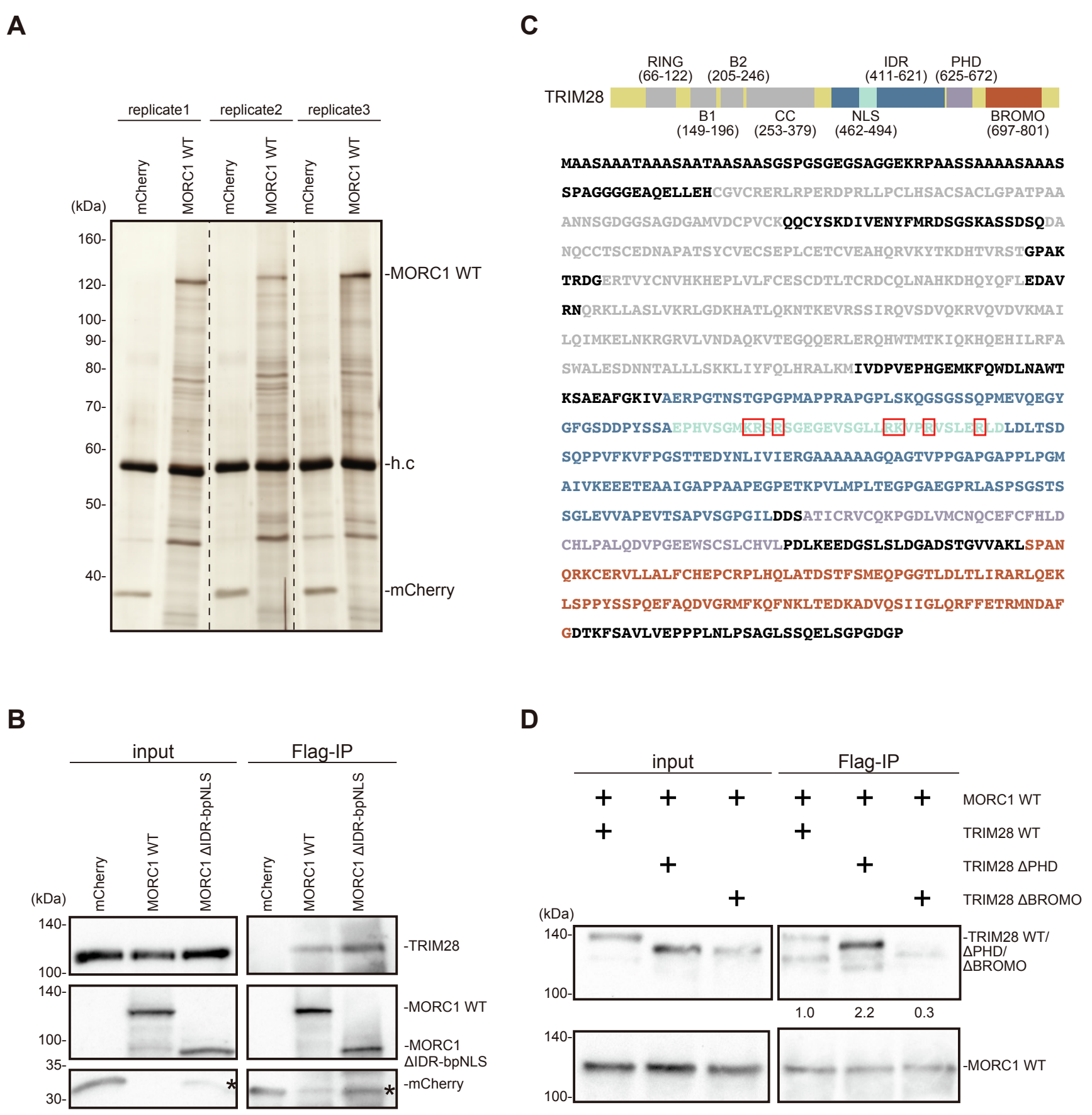

Figure S2. MORC1 interaction with TRIM28 and mapping of TRIM28 domains in NIH3T3 cells.

(A) Silver-stained SDS-PAGE showing proteins immunoprecipitated with Flag antibody from NIH3T3 cells expressing 3xFlag-mCherry (control) or 3xFlag-MORC1 WT. **(B)** Co-immunoprecipitation of exogenously expressed MORC1 WT or ΔIDR-bpNLS and endogenously expressed TRIM28 in NIH3T3 cells. Asterisks indicate degradation products. **(C)** Schematic representation of TRIM28 domains and the amino acid sequence, with sequence regions colored to match the domains in the schematic. Basic residues within the NLS (green) are highlighted with red boxes. **(D)** Co-immunoprecipitation of exogenously expressed MORC1 WT and exogenously

expressed TRIM28 WT or ΔPHD or ΔBROMO in NIH3T3 cells. The signal for co-immunoprecipitated TRIM28 WT, ΔPHD, or ΔBROMO was normalized to the signal for immunoprecipitated MORC1. This value was further normalized to the TRIM28 WT, ΔPHD, or ΔBROMO signal in the input samples, expressed relative to the WT value, and indicated below the blot.

A

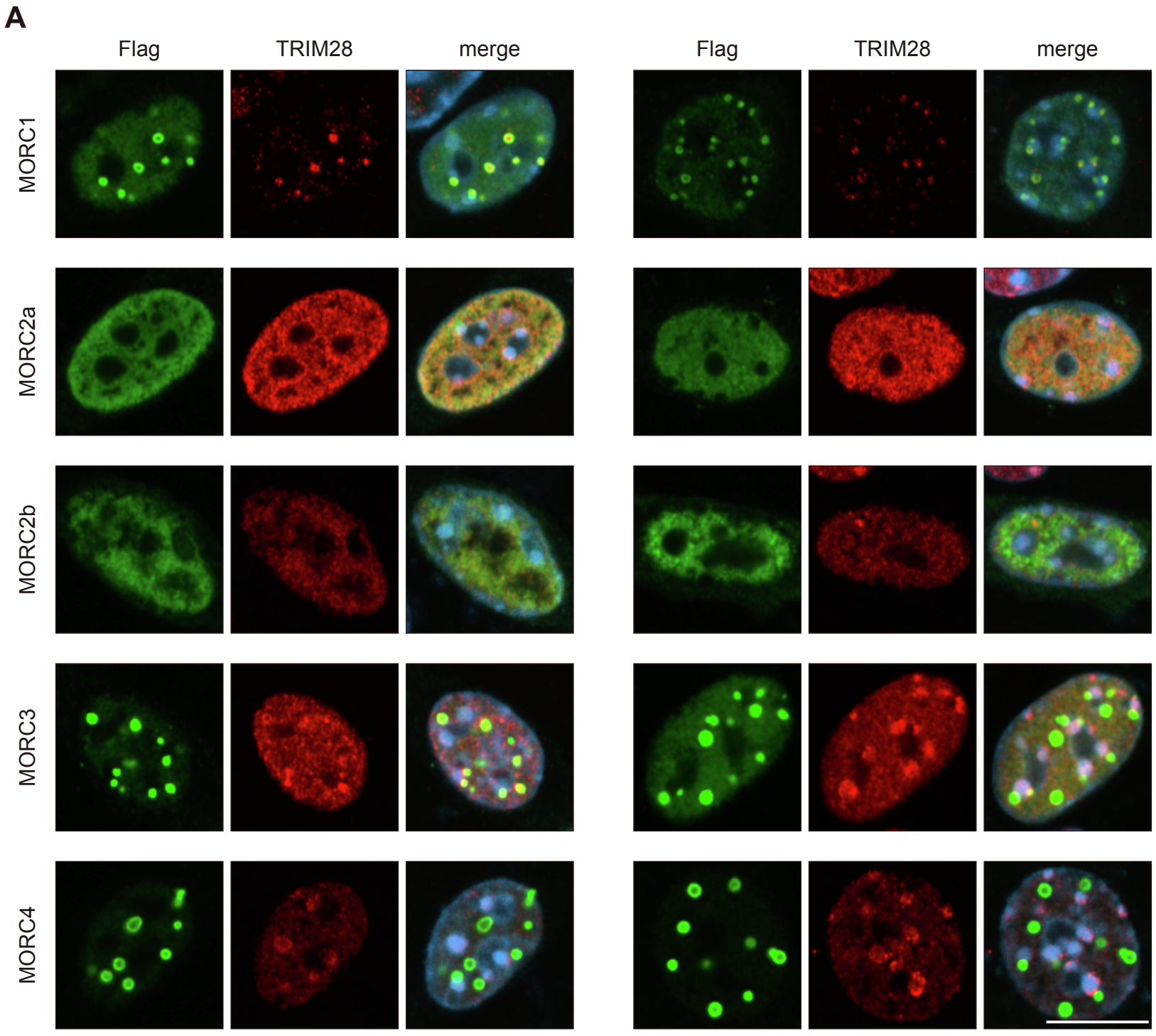

B

```
MORC1 470 QWRRRQAMGIPFILQCDLCLKWRVLPSSSNYQEKGLPDLWICASNPNNLENSCNQIERLPSIP 532
MORC3 401 DI---QKRPDQTWVQCDACLKWRKLPDGI----DQLPEKWYCSNNPDPQFRNCEVPEEPEDED 456
```

Figure S3. Subcellular localization of MORC family members and TRIM28 in NIH3T3 cells, and CW domain alignment between MORC1 and MORC3.

(A) Subcellular localization of MORC family members in NIH3T3 cells (green). Subcellular localization of TRIM28 is also shown (red). Nuclei stained with DAPI (blue). Scale bar, 10 μm. **(B)** Amino acid sequence alignment of the CW domain from MORC1 and MORC3. Residues that are identical between the two proteins are highlighted in yellow.

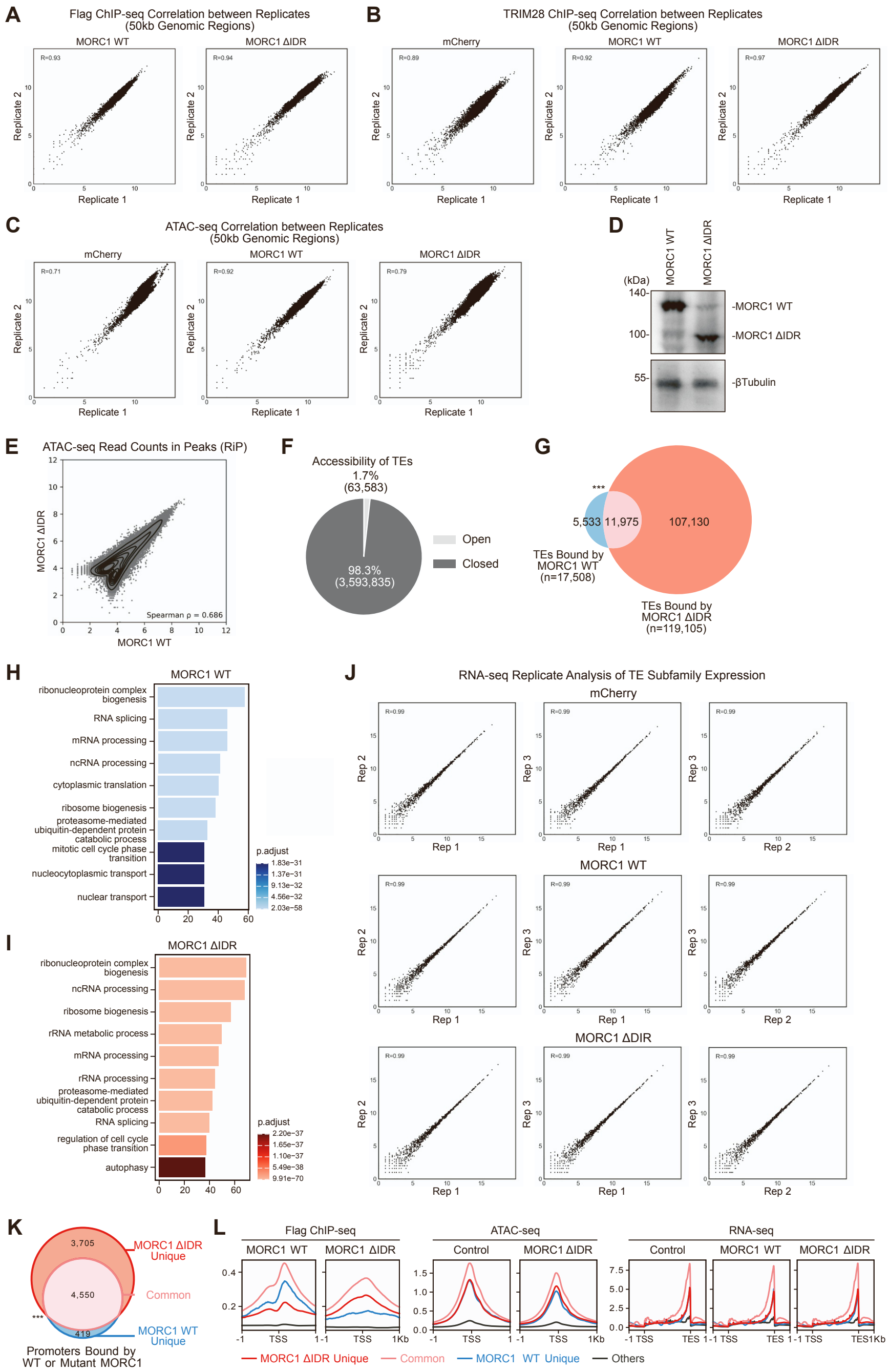

A
Flag ChIP-seq Correlation between Replicates
(50kb Genomic Regions)
MORC1 WT
MORC1 ΔIDR
B
TRIM28 ChIP-seq Correlation between Replicates
(50kb Genomic Regions)
mCherry
C
ATAC-seq Correlation between Replicates
(50kb Genomic Regions)
D
-MORC1 WT
-MORC1 ΔIDR
-βTubulin
E
ATAC-seq Read Counts in Peaks (RiP)
Spearman ρ = 0.686
F
Accessibility of TEs
1.7%
(63,583)
98.3%
(3,593,835)
Open
Closed
G
5,533
11,975
107,130
TEs Bound by
MORC1 WT
(n=17,508)
TEs Bound by
MORC1 ΔIDR
(n=119,105)
H
ribonucleoprotein complex biogenesis
RNA splicing
mRNA processing
ncRNA processing
cytoplasmic translation
ribosome biogenesis
proteasome-mediated ubiquitin-dependent protein catabolic process
mitotic cell cycle phase transition
nucleocytoplasmic transport
nuclear transport
p.adjust
I
rRNA metabolic process
rRNA processing
regulation of cell cycle phase transition
autophagy
J
RNA-seq Replicate Analysis of TE Subfamily Expression
MORC1 ΔDIR
K
3,705
4,550
419
MORC1 ΔIDR Unique
Common
MORC1 WT Unique
Promoters Bound by
WT or Mutant MORC1
L
Flag ChIP-seq
ATAC-seq
RNA-seq
Control
MORC1 ΔIDR Unique
Common
MORC1 WT Unique
Others

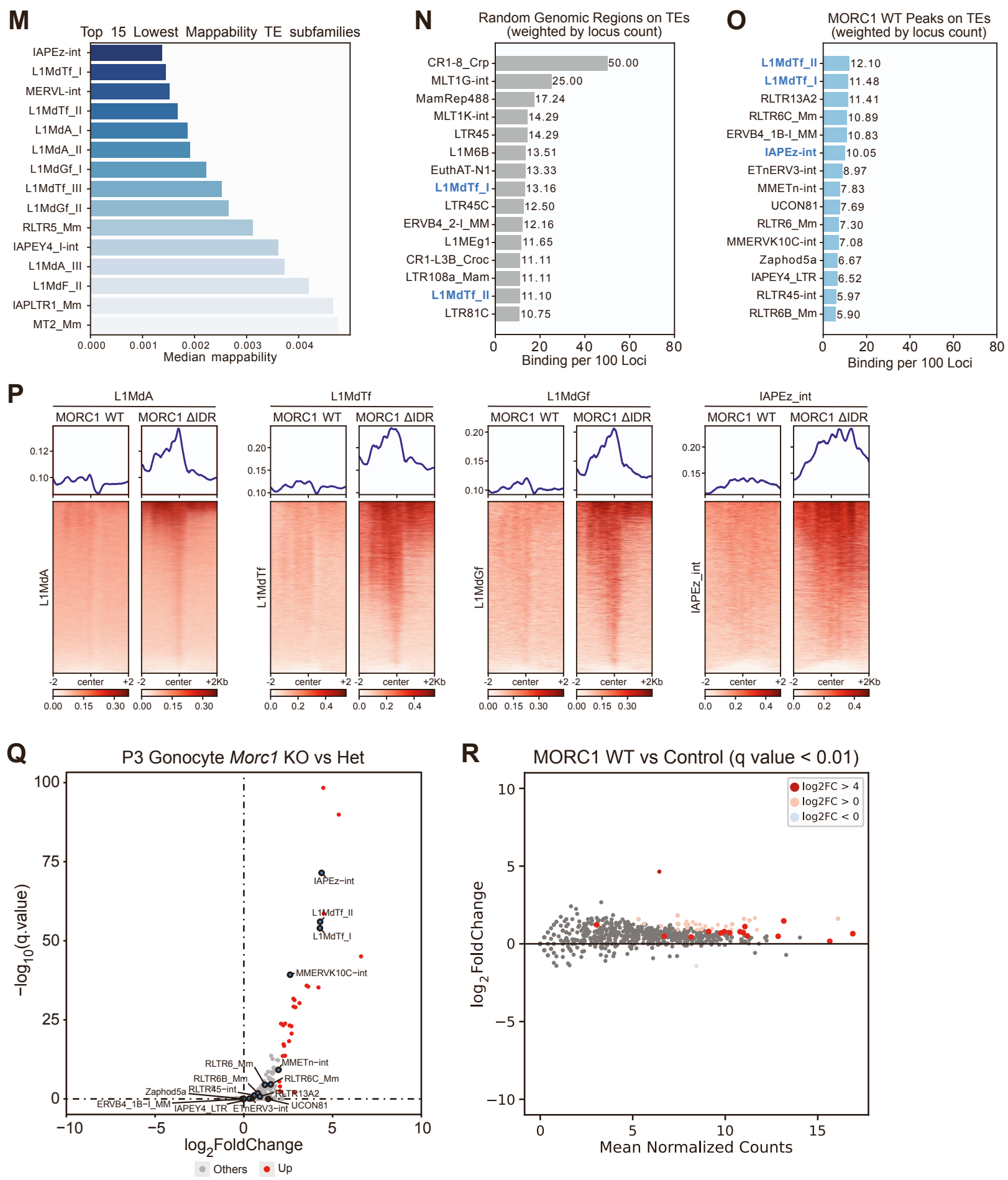

Figure S4. IDR-dependent control of MORC1 genomic occupancy.

(A–C) Correlation between biological replicates for Flag ChIP-seq (A), TRIM28 ChIP-seq (B), and ATAC-seq (C). Read counts within 50-kb genomic bins were used for the analysis. Spearman correlation coefficients are indicated. **(D)** Western blot showing Dox-induced expression of MORC1 WT and ΔIDR in NIH3T3 cells. β-Tubulin, control. **(E)** Scatter plot comparing ATAC-seq read counts in universal peaks recognized in NIH3T3 cells expressing MORC1 WT (x-axis) and ΔIDR (y-axis). Spearman correlation coefficient is indicated. **(F)** Proportion of transposons classified as open or closed based on overlap with ATAC–seq peaks in NIH3T3 cells (mCherry). **(G)** Venn diagram

showing the overlap between transposons bound by MORC1 WT and MORC1 ΔIDR. *** indicates a significant enrichment of overlap relative to random expectation, as determined by a one-sided Fisher's exact test ($P < 0.001$). **(H, I)** Bar plots showing Gene Ontology enrichment for genes with promoters bound by MORC1 WT (H) and MORC1 ΔIDR (I). **(J)** Correlation between biological replicates for RNA-seq. Read counts of transposon subfamilies were used for the analysis, and Spearman correlation coefficients are indicated. **(K)** Venn diagram showing the overlap between promoters bound by WT and mutant MORC1 in NIH3T3 cells. *** indicates a significant enrichment of overlap relative to random expectation, as determined by a one-sided Fisher's exact test ($P < 0.001$). **(L)** Line plots showing signal profiles across promoter or gene regions for each category and the "others" group. Left, Flag ChIP-seq in NIH3T3 cells expressing MORC1 WT or ΔIDR. Middle, ATAC-seq in control (mCherry) and MORC1 ΔIDR-expressing NIH3T3 cells. Right, RNA-seq in control (mCherry), MORC1 WT-expressing, and MORC1 ΔIDR-expressing NIH3T3 cells. **(M)** Top 15 evolutionarily young transposon subfamilies in mice, defined by the median mappability of each subfamily. **(N)** Top 15 transposon (TE) subfamilies most frequently overlapped by random genomic peaks generated by shuffling MORC1 ΔIDR peaks while preserving peak number and length. Binding frequency was normalized per 100 loci. Evolutionarily young subfamilies indicated in (M) are highlighted in blue. **(O)** Top 15 transposon (TE) subfamilies most frequently overlapped by MORC1 WT peaks, with binding frequency normalized per 100 loci. Evolutionarily young subfamilies indicated in (M) are highlighted in blue. **(P)** Heatmap showing MORC1 WT and ΔIDR enrichment across representative young transposon subfamilies listed in Figure 4F. For each sample, merged files from two biological replicates were used for the analysis. **(Q)** Volcano plot of transposon expression changes in P3 gonocytes from *Morc1* knockout (KO) and heterozygous (Het)

mice. Significantly upregulated transposon subfamilies are highlighted in red, and subfamilies listed in (O) are annotated. **(R)** MA plot showing expression changes in transposon subfamilies between NIH3T3 cells expressing MORC1 WT and control (mCherry). Subfamilies with q.value < 0.01 and meeting different log2FC thresholds are annotated as indicated in the legend. Subfamilies listed in (O) are highlighted in bright red.

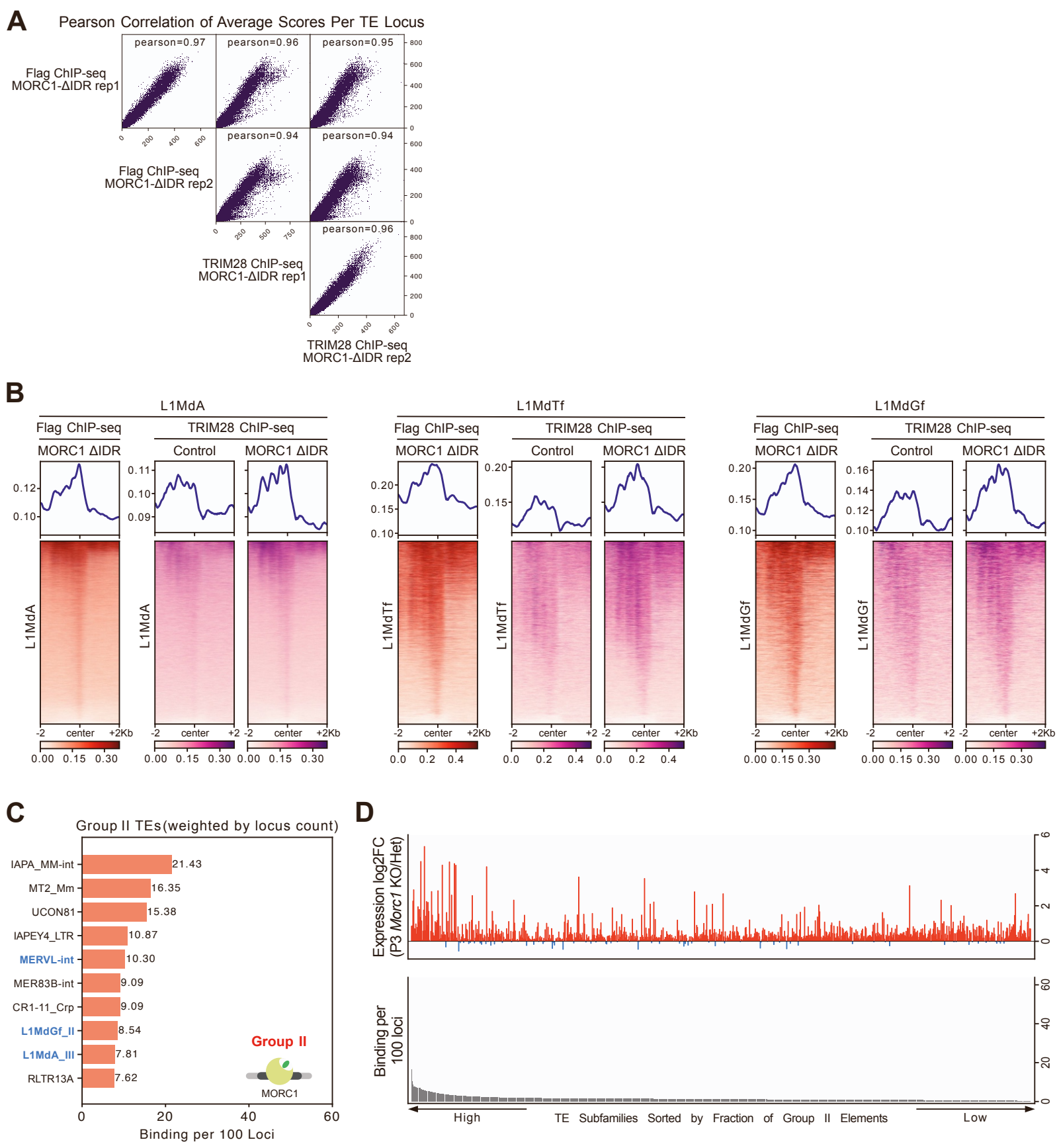

Figure S5. MORC1 modulates TRIM28 recruitment to target transposons.

(A) Pairwise correlations between biological replicates of MORC1-ΔIDR Flag ChIP-seq and TRIM28 ChIP-seq across filtered TE loci. Pearson correlation coefficients are indicated. **(B)** Heatmap showing MORC1 ΔIDR enrichment and TRIM28 enrichment in control (mCherry) and MORC1 ΔIDR-expressing NIH3T3 cells across representative young transposon subfamilies shown in Figure 5E. For each sample, merged files from two biological replicates were used for the analysis. **(C)** Top 10 transposon (TE) subfamilies within Group II, with binding frequency normalized per 100 loci. Evolutionarily young subfamilies indicated in Figure S4M are highlighted in blue. **(D)**

Correlation within transposon subfamilies between the fraction of Group II transposons (bottom) and expression changes in P3 *Morc1*-deficient gonocytes (top).
